# Integrator complex removes lesion-stalled RNA polymerase II after transcription-coupled repair to facilitate transcription restart

**DOI:** 10.1093/nar/gkag1068

**Published:** 2026-10-31

**Authors:** Cindy M A Blom, Emilio Gómez-Vázquez, Marjolein van Sluis, Jurgen A Marteijn

**Affiliations:** Department of Molecular Genetics, Oncode Institute, Erasmus MC Cancer Institute, Erasmus University Medical Center, 3015 GD,Rotterdam, The Netherlands; Department of Molecular Genetics, Oncode Institute, Erasmus MC Cancer Institute, Erasmus University Medical Center, 3015 GD,Rotterdam, The Netherlands; Department of Molecular Genetics, Oncode Institute, Erasmus MC Cancer Institute, Erasmus University Medical Center, 3015 GD,Rotterdam, The Netherlands; Department of Molecular Genetics, Oncode Institute, Erasmus MC Cancer Institute, Erasmus University Medical Center, 3015 GD,Rotterdam, The Netherlands

## Abstract

Gene transcription by RNA Polymerase II (Pol II) is highly vulnerable to transcription-blocking DNA lesions (TBLs), which, if unresolved, induce transcription stress leading to cellular decline and aging. TBLs are removed by transcription-coupled nucleotide excision repair (TC-NER). TC-NER is initiated when Cockayne syndrome group B protein recognizes lesion-stalled Pol II and promotes TC-NER complex assembly. This complex recruits TFIIH for lesion verification, followed by incision and repair to enable transcription restart. Many TBLs stall Pol II within its active site, shielding the lesion from repair and requiring Pol II processing to expose it. However, whether this occurs through Pol II backtracking or release from the DNA, and the regulatory mechanisms involved, remain poorly defined. Here, we identified the Integrator complex as a key factor in lesion-stalled Pol II processing. Interaction proteomics revealed Integrator recruitment to late-stage TC-NER complexes. Integrator’s endonuclease core promotes efficient transcription recovery after TBL induction, without affecting lesion removal by TC-NER. Live-cell imaging and chromatin retention assays demonstrated that Integrator facilitates Pol II release from DNA following TC-NER. Collectively, these findings support a two-step mechanism for Pol II clearance during TC-NER, wherein TFIIH-mediated backtracking exposes the lesion for repair, followed by Integrator-mediated Pol II release to restore transcription.

## Graphical Abstract

**Graphical Abstract:**
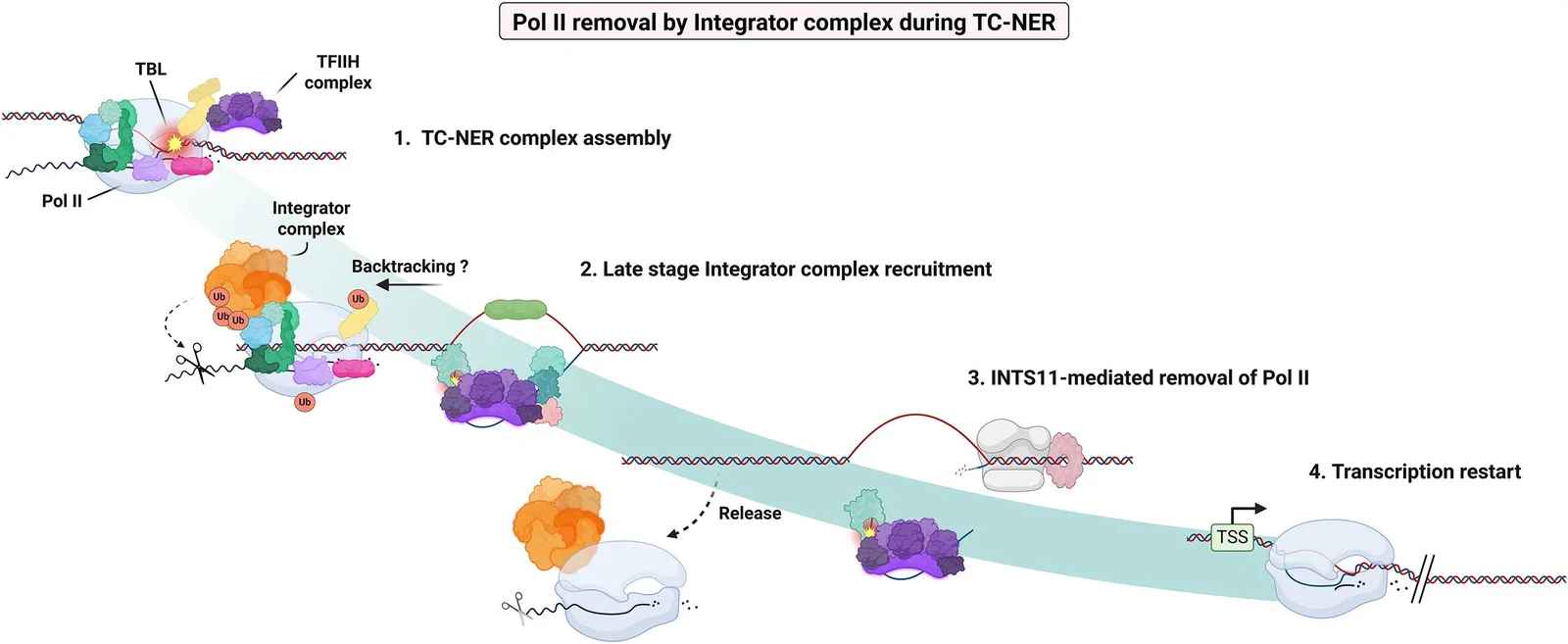

## Introduction

RNA polymerase II (Pol II)-mediated transcription is essential for maintaining cellular integrity and proper cell function. However, elongating Pol II is vulnerable to DNA damage that impedes or completely stalls its forward progression [1–4]. These transcription-blocking DNA lesions (TBLs) arise from endogenous sources such as aldehydes and environmental factors such as ultraviolet (UV) light [4–6]. Failure to properly resolve TBLs cause transcription stress that drives cellular decline, senescence, and apoptosis associated with aging [2, 6, 7]. An important pathway to remove these cytotoxic TBLs is transcription-coupled nucleotide excision repair (TC-NER), which resolves a broad range of bulky DNA lesions from the transcribed strand of active genes, such as UV light-induced photoproducts, intrastrand crosslinks, and DNA–protein crosslinks (DPCs) [2, 8–12]. The importance of TC-NER is underscored by genetic disorders such as the Cockayne syndrome, which is caused by inactivating mutations in TC-NER genes and characterized by progressive neurodegeneration and premature aging [13].

TC-NER is initiated by Cockayne syndrome group B protein (CSB), which can discriminate between damage-stalled Pol II and other forms of transcriptional-paused Pol II (e.g. natural pause sites) [4]. CSB facilitates Pol II bypass by its DNA translocase activity at damage-free blocks. However, when bypass fails due to bulky DNA damage, CSB remains stably bound to lesion-stalled Pol II, thereby initiating TC-NER [14–16] through recruitment of CSA and UVSSA to assemble the full TC-NER complex [17–19]. CSA is recruited as part of the Cullin–RING ubiquitin ligase complex CRL4^CSA^ [17, 20, 21], which ubiquitylates Pol II [22, 23] and the TC-NER factors CSB [21] and UVSSA [18, 22, 24]. Pol II ubiquitylation, which promotes progression of TC-NER by facilitating UVSSA and TFIIH recruitment [22], is supported by ELOF1 [25, 26] and the recently identified TC-NER factor STK19, which stimulates CRL4^CSA^ stability [27]. UVSSA promotes the recruitment of TFIIH by a direct interaction with the p62 subunit of TFIIH [17, 28], and TFIIH recruitment is further stimulated by the UVSSA ubiquitylation at lysine 414 [22, 24]. STK19 plays an important role in the correct positioning of TFIIH via a direct interaction with XPD [27, 29–32]. TFIIH, together with XPA and RPA, unwinds the DNA. Following damage verification, the TBL is excised by the structure-specific endonucleases ERCC1-XPF and XPG through dual incision [33, 34]. The TBL containing DNA fragment is subsequently evicted in a process that is stimulated by HLTF [35]. The resulting single-stranded DNA (ssDNA) gap is repaired by DNA polymerases and sealed by ligation, allowing transcription to restart [2].

Most TBLs, including UV-induced lesions such as cyclobutane pyrimidine dimers (CPDs) and 6–4 photoproducts (6–4PPs), stall Pol II when they reach the active site of Pol II, generating a highly stable complex with an *in vitro* half-life of ∼20 h [36–38]. As a consequence, these TBLs become fully enclosed within the Pol II elongation complex, and this steric shielding of Pol II prevents proofreading and excision of the TBL by the incision complex consisting of TFIIH, XPA, RPA, XPF/ERCC1, and XPG [2, 39]. To permit repair, lesion-stalled Pol II must therefore be processed to expose the TBL to the downstream incision complex. However, how exactly Pol II is processed to hand-over the TBL from the TC-NER complex to the incision complex, and which factors are involved, remains largely unknown. Several non-mutually exclusive models have been proposed. One model proposes that Pol II is backtracked (reverse translocated), thereby exposing the TBL, while Pol II remains bound to the DNA template. Biochemical and structural studies in bacteria demonstrated that the helicase UvrD promotes RNA polymerase backtracking to expose transcription-blocking lesions for repair [40]. As backtracking is a conserved feature of cellular RNA polymerases, a similar mechanism has been proposed for mammalian TC-NER [2, 3, 39, 41, 42]. A likely candidate to facilitate this backtracking during TC-NER in mammals is TFIIH. Recent cryo-EM models [27, 29, 30] suggest that the translocase XPD subunit of TFIIH is optimally positioned to use its 5′-3′ translocase activity to pull the TBL out of the Pol II active site toward TFIIH, thereby pushing Pol II backward. Alternatively, lesion-stalled Pol II might be actively released from TBLs due to premature transcription termination [43–45]. However, it remains unclear whether this happens during TC-NER to provide the incision machinery access to the lesion, or at a later moment as a consequence of repair. Pol II might also be cleared from the TBL by degradation [23]. However, Pol II degradation is most likely not the primary mechanism during repair, as the ubiquitin dependent segregase VCP/p97, which mediates chromatin extraction and degradation of ubiquitylated Pol II [46–48], is dispensable for TC-NER and for removal of lesion-stalled Pol II in TC-NER proficient cells [49]. Instead, VCP/p97 facilitates extraction and degradation of lesion-stalled Pol II when TC-NER is impaired [19, 49, 50], as for example in UVSSA deficient cells where Pol II is still ubiquitylated by CRL4^CSA^, indicating that proteasomal Pol II clearance serves primarily as a fail-safe mechanism in TC-NER limiting conditions [2, 22, 50, 51].

In this study, we identified the Integrator complex as important factor regulating the release of lesion-stalled Pol II during the cellular response to transcription stress, in line with a previous multi-omics study that implicated the Integrator complex in that pathway [52]. The Integrator complex is a multifunctional regulator of Pol II transcription, composed of 15 subunits (INTS1–15) forming an ∼1.5 MDa complex that binds the Pol II C-terminal domain (CTD) [53, 54]. Initially identified for 3′-end processing of U-rich small nuclear RNAs (snRNAs) [53], Integrator is now recognized as a broad regulator of transcription, controlling both protein-coding and non-coding RNAs [55, 56]. A key function of Integrator is RNA cleavage, mediated by its endonuclease module composed of INTS4, INTS9, and INTS11, the latter providing the catalytic activity [57–59]. This endonuclease activity not only processes snRNAs, but also cleaves nascent mRNAs to induce premature transcription termination at promoter-proximal and early elongation sites, or later during elongation when transcription is stalled or non-productive [60]. In addition, Integrator contains a serine/threonine-protein phosphatase 2A (PP2A) module, consisting of a PP2A heterodimer tethered by INTS5, INTS6, and INTS8, which dephosphorylates pSer2 on the Pol II CTD and associated elongation factors such as DSIF [61]. The Int-PP2A module of Integrator counteracts CDK9 activity, which normally facilitates the transition of paused Pol II into productive elongation [62]. This modulation occurs at many protein-coding genes, thereby fine-tuning promoter-proximal pausing and transcriptional elongation [61]. Through its endonuclease and phosphatase activities, Integrator broadly regulates transcriptional pausing, termination, and stress responses [63–66], underscoring its central role in maintaining transcriptional homeostasis.

Here, we show that the Integrator complex plays a crucial role in the cellular response to DNA damage-induced transcription stress by promoting the removal of lesion-stalled Pol II. We identified the Integrator complex as a DNA damage-induced interactor of late-stage TC-NER complexes. However, the Integrator complex is dispensable for the repair of TBLs. Instead, the Integrator complex facilitates eviction of Pol II after repair has taken place, to enable efficient transcription recovery. Together, these findings support a two-step model of Pol II clearance: first, Pol II backtracking exposes the lesion for repair, after which the Integrator complex releases the previously lesion-stalled Pol II from chromatin, allowing transcription to restore through *de novo* transcription initiation.

## Materials and methods

### Cell lines and cell culture

All HCT116 colorectal cancer cells, RPE1 retinal pigment epithelium cells, and Hela human cervical carcinoma cells were cultured in Dulbecco’s modified Eagle’s medium (DMEM; Gibco) supplemented with 10% fetal bovine serum (FBS; Gibco) and 1% penicillin–streptomycin (Gibco) at 37°C with 5% CO₂ in a humidified incubator. HCT116 GFP–RPB1 knock-in (KI), RPE1 GFP–RPB1 KI [12, 67], RPE1 GFP-RPB1 KI XPC knockout (KO) [12], HCT116 CSB–mScarletI KI [25, 68], and TC-NER KO [25] cells were generated as described.

The Halo-HA-INTS11 repair templates for CRISPR/Cas9 mediated gene-editing was ordered at Genscript in a pUC57 plasmid. The repair template contained two homology arms (200 bp) surrounding the INTS11 start codon, Halo-HA-tag followed by an 81-bp linker to link it in frame with the start codon of INTS11. Four silent mutations at the PAM site were introduced to avoid Cas9 re-cleavage.

HCT116 and RPE1 cells were co-transfected with Cas9 protein, pre-incubated with sgRNA (crINTS11: 5′-CGGCGGCTCCCACTCACCCA-3′) targeting the N-terminal domain of INTS11 and the designed HDR template using Lipofectamine CRISPRMAX Cas9 reagent (Invitrogen) for HCT116 cells or nucleofection with a 4D Nucleofector (Lonza, V4XC-1024) for RPE1 cells, according to the manufacturer’s instructions. Following transfection, cells were cultured for 2 days in medium supplemented with 2 nM NU7441 (DNA-PK inhibitor; Selleckchem) and 10 μM ART558 (Polθ inhibitor; MedChemExpress). Halo-HA KI cells were enriched through two successive rounds of fluorescence-activated cell sorting (FACS) using the JFx554 Halo-ligand (500 nM, Levis Lab) to stain cells containing a Halo tag. Cells were incubated with the ligand in DMEM for 15 min at 37°C, washed three times with phosphate buffered saline (PBS), and further incubated in ligand-free medium for 30 min. After three additional PBS washes, cells were incubated in fresh medium for 1 h, washed once with PBS, harvested, and the resulting pellet was washed twice with PBS. Finally, the pellet was resuspended in PBS containing 10% FBS for FACS enrichment. Single cells were seeded to establish clonal populations and expanded for genotyping by polymerase chain reaction on genomic DNA with the primers FW 5′-TCAGAAAGATGCAGTCCCATAG-3′, RV1 5′-GTAGTGAGGATCGAAGGGAAAG-3′, and RV2 5′-AGGTCAAGGCACCGACGA-3′. Correct KI was confirmed by Sanger sequencing. Positive clones were further validated by immunoblotting.

Halo-HA-INTS11 was depleted using Halo-PROTAC3 (referred to as PROTAC3 throughout the manuscript) (MedChemExpress, MCE) mediated degradation. PROTAC3 was used at a final concentration of 0.3 µM, otherwise the used concentration is indicated. Unless otherwise noted, pre-treatments lasted 16 h and were kept for the duration of the whole experiment.

For siRNA transfection, cells were transfected twice with 6 h intervals, using 6 μl Lipofectamine RNAi Max (Invitrogen) and 6 μl siRNA (20 μM stock). Cells were transfected 36–48 h prior to harvesting/functional experiments according to the manufacturer’s instructions. The following siRNAs were acquired from Horizon Discovery: siINTS11 #1: 5′-GACAGACCCUGGGCCUGCA-3′; siINTS11 #2: 5′- CAGACUUCCUGGACUGUGU-3′; siCSB: 5′-GCAUGUGUCUUACGAGAUA-3′; and siCTRL: 5′-UGGUUUACAUGUUGUGUGA-3′. INTS9 and INTS11-stably expressed shRNAs [55, 64] were induced with doxycycline (Dox; 1 µg/ml) for 24 h prior to start of experiments, and subsequently maintained in Dox-containing medium during the experiment. Lentiviral expression constructs encoding FLAG-tagged WT INTS11 or the catalytically inactive mutant INTS11 (E203Q) [55, 64, 69] were transduced into RPE1 Halo-RPB1 KI cells. Following transduction, cells were selected with 10 μg/ml puromycin. Expression of the transgenes was verified by immunofluorescence (IF) using an anti-FLAG antibody.

### Treatment with DNA-damaging agents and inhibitors

UV-C DNA damage was induced by irradiating cells with the indicated UV doses using a 254-nm germicidal UV-C lamp (Philips) after washing with PBS and removal of all liquids. Exposure time was controlled by an air-pressured shutter connected to a timer. For chemical genotoxins, cells were treated with Illudin S (75 ng/ml; I019, TOKU-E) for 2 h, or with trabectedin (30 nM, Sigma) for 2 h in culture medium. Formaldehyde-induced damage was introduced by exposing cells to 1 mM formaldehyde (FA, Pierce, 16% methanol-free solution, Thermo Scientific) for 30 min, after which the compound was quenched by three washes in culture medium for recovery of RNA synthesis (RRS). For Pol II FRAP, cells were exposed to 300 µM FA for 30 min before imaging and FA was kept on the cells during imaging.

For reversible transcription inhibition, cells were incubated with flavopiridol (2 μM; Sigma) for 2.5 h. Ribosomal activity was inhibited with cycloheximide (100 μM; Sigma), added 1 h prior to treatments, and maintained throughout the experiment. NEDDylation was inhibited with NAE inhibitor MLN4924 (10 µM; Sigma) 30 min before treatment. XPB was degraded with spironolactone (10 µM, Sigma, 2 h).

### Proteomics

For on-gel digestion, sodium dodecyl sulfate–polyacrylamide gel electrophoresis (SDS–PAGE) gel lanes were cut into 2-mm slices and subjected to in-gel reduction with dithiothreitol, alkylation with chloroacetamide, and digested with trypsin (sequencing grade; Promega), as described previously [18].

Mass spectrometry was performed on a Vanquish Neo UHPLC system coupled to an Eclipse Orbitrap (for ubiquitinome analysis) or on an EASY-nLC 1200 LC system coupled to an Orbitrap Fusion Lumos Tribrid (for interactome analysis) mass spectrometer (all Thermo Fisher Scientific), operating in positive mode and equipped with a nanospray source. Peptide mixtures were trapped on a ReproSil C18 reversed phase column (Dr Maisch GmbH; column dimensions 1.5 cm × 100 µm, packed in-house) at a flow rate of 8 μl/min. Peptide separation was performed on ReproSil C18 reversed phase column (Dr Maisch GmbH; column dimensions 15 cm × 50 µm, packed in-house) using a linear gradient from 0% to 80% B [A = 0.1% FA; B = 80% (v/v) AcN, 0.1% FA] in 70 or 120 min and at a constant flow rate of 200 nl/min. The column eluent was directly sprayed into the ESI source of the mass spectrometer. All spectra were recorded in data dependent acquisition mode and MS1 full scans were recorded at a resolution of 120 000 in the scan range from 350 to 1650 *m*/*z* with a maximum injection time set to 50 ms (AGC target: 4E5). For MS2 acquisition, HCD with an NCE of 28% and an isolation window of 1.6 Da were used and MS2 scans were recorded in the ion trap. For the ubiquitinome analysis, a single LC-MS/MS run was performed for all immunoprecipitated peptide material from one sample.

Raw mass spectrometry data were analyzed with the MaxQuant software suite [70] (version v.1.6.3.3) as described previously [18]. A false discovery rate of 0.01 for proteins and peptides and a minimum peptide length of 7 amino acids were set. The Andromeda search engine was used to search the MS/MS spectra against the Uniprot database (taxonomy: *Homo sapiens*, release 2018) concatenated with the reversed versions of all sequences. A maximum of two missed cleavages was allowed. The peptide tolerance was set to 10 ppm and the fragment ion tolerance was set to 0.6 Da for HCD spectra. The enzyme specificity was set to trypsin and cysteine carbamidomethylation was set as a fixed modification (except for the ubiquitinome analysis). MaxQuant automatically quantified peptides and proteins based on standard SILAC settings (multiplicity = 2, K8/R10). SILAC protein ratios were calculated as the median of all peptide ratios assigned to the protein. In addition, a posterior error probability for each MS/MS spectrum ≤0.1 was required. In case the identified peptides of two proteins were the same or the identified peptides of one protein included all peptides of another protein, these proteins were combined by MaxQuant and reported as one protein group. Before further statistical analysis, known contaminants and reverse hits were removed.

### Cell lysis and immunoblotting (Western blot)

Total cell lysates were harvested by directly scraping cells after PBS washes in SDS–PAGE sample buffer (125 mM Tris, pH 6.8, 2% SDS, 21% glycerol, 0.005% bromophenol blue, and 4% β-mercaptoethanol). Benzonase Nuclease (Merck Millipore) 10 U/ml was added to the total cell lysate to degrade genomic DNA, and samples were incubated on ice for 50 min. Lysates were boiled at 95°C for 5 min prior to separation by SDS–PAGE using 4%–15% Mini-Protean TGX precast protein gel (Bio-Rad) in running buffer (1.44% (w/v) glycine, 0.3% (w/v) Tris, 0.1% (w/v) SDS), using the Precision Plus all blue standards (Bio-Rad) for molecular weight estimation.

Proteins were transferred onto hydrophobic polyvinylidene difluoride membranes (0.45 µm PVDF, Merck Millipore) at 30 V overnight (o/n) at 4°C in transfer buffer (25 mM Tris, 192 mM glycine, and 10% ethanol, or 25 mM Tris, 192 mM glycine, and 20% ethanol for chromatin fractionation) in a Biorad system. Membranes were blocked for 1 h at room temperature (RT) in 5% bovine serum albumin (BSA; Sigma) (or 3% BSA for chromatin fractionation) in PBS-Tween (0.05%), followed by incubation with primary antibody for 2 h at RT or 4°C o/n. Membranes were washed 3× for 10 min in 0.05% PBS-T after each antibody incubation. IR-Dyes conjugated secondary antibodies (Sigma) were incubated for 1 h at RT and detected using an Odyssey CLx infrared scanner (LI-COR). Image Studio Lite (v.6.0) was used for Western blot acquisition and analysis. Primary and secondary antibodies are listed below (Table 1).

**Table 1.** Primary and secondary antibody list and working solutions

Antibody	Host	Source	WB dilution	IF dilution
CSB/ERCC6	Rabbit	Antibodies-online, ABIN2855858	1:1000	N.A.
CSA/ERCC8	Mouse	Santa Cruz, sc376981	1:500	N.A.
CSA/ERCC8	Rabbit	Abcam, ab240096	1:1000	N.A.
pSer2-Pol II	Rabbit	Abcam, ab5095	1:1000	1:1000
Tubulin (clone B512)	Mouse	Sigma, T5168	1:1000	N.A.
XPB (clone 15TF2-1B3)	Mouse	Sigma, MABE1123	1:1000	N.A.
XPD	Mouse	Abcam, ab54676	1:1000	N.A.
INTS11	Rabbit	Sigma, HPA029025	1:1000	N.A.
INTS9	Rabbit	Sigma, HPA051615	1:1000	1:1000
HA	Rat	Roche, 11867423001	1:1000	1:1000
ATF3	Rabbit	Cell Signaling, 33593S	1:1000	N.A.
CPDs	Mouse	Cosmo Bio, CAC-NM-DND-001	N.A	1:1000
BRG1	Rabbit	Abcam, ab110641	1:1000	N.A
Anti-mouse Alexa Fluor 488	Goat	Thermo Fisher, A-11029	N.A	1:1000
Anti-rabbit Alexa Fluor 555	Goat	Thermo Fisher, A-21429	N.A	1:1000
Anti-rat Alexa Fluor 680	Goat	Thermo Fisher, A-21096	N.A	1:1000
Anti-rabbit CF IRDye 770	Goat	Sigma, Sab4600215	1:10 000	N.A.
Anti-rabbit CF IRDye 680	Goat	Sigma, Sab4600200	1:10 000	N.A.
Anti-mouse CF IRDye 680	Goat	Sigma, Sab4600199	1:10 000	N.A.
Anti-Rat CF IRDye 770	Goat	Sigma, Sab4600479	1:10 000	N.A.

### Chromatin fractionation

Cells grown in six-well plates were lysed in fractionation buffer B1 (30 mM HEPES, pH 7.6, 1 mM MgCl₂, 150 mM NaCl, 0.5% NP-40, 50 μM MG132 (Enzo), complete EDTA-free protease inhibitor cocktail (Roche), phosphatase inhibitor cocktail II (Sigma), and N-ethylmaleimide (Sigma)) for 20 min on ice. Lysates were centrifuged at 10 000 × *g* for 5 min at 4°C to pellet chromatin, and the supernatant was discarded. The pellet was resuspended in 25 μl buffer B1 and treated with 25 U Benzonase (Millipore) to digest the chromatin, and incubated for 1 h at 4°C. Following centrifugation at maximum speed for 10 min at 4°C, the resulting supernatant was mixed with 25 μl Laemmli sample buffer, and boiled for 5 min at 95°C. Proteins were resolved using 4%–15% MiniProtean TGX precast protein gels (Bio-Rad) and analyzed by immunoblotting.

### Immunoprecipitation

Immunoprecipitation (IP) was performed as described [17, 25, 27] on mock or UV-C (16 J/m^2^) (± PROTAC3) treated cells. In brief, cells on three confluent 14.5 cm dishes per condition were harvested by trypsinization and collected in medium. Next, cells were washed with PBS and collected by centrifugation for 5 min at 1000 rpm. The cell pellets were resuspended and lysed in buffer B1 (30 mM HEPES, pH 7.6, 1 mM MgCl_2_, 150 mM NaCl, 0.5% NP-40, EDTA-free Protease Inhibitor Cocktail (Roche), N-ethylmaleimide crystalline, 50 μM proteasome inhibitor MG132 (Enzo), and phosphatase cocktail II (Sigma)) and incubated for 20 min at 4°C. Chromatin was pelleted by centrifugation for 5 min at 10 000 × *g* at 4°C and the supernatant was discarded afterward. Thereafter, chromatin was digested in buffer B1 containing 500 U benzonase (Millipore) for 1 h at 4°C.

For Pol II IP, 2 µg pSer2-modified Pol II antibody (ab5095, Abcam) was added during the benzonase treatment. The undigested fraction was pelleted by centrifuging at 13 000 rpm for 10 min at 4°C. The soluble antibody-bound fraction was incubated with salmon sperm protein A agarose beads (Millipore) for 1.5–2 h. For Halo-HA-INTS11 IP, the soluble fraction was incubated with 25 μl monoclonal anti-HA-agarose antibody beads (Millipore) for 1.5–2 h at 4°C, and Protein G agarose beads (Pierce) were used for binding control samples. Monoclonal anti-HA-agarose beads and Protein G agarose beads were washed and incubated with BSA and salmon sperm DNA before use in IP. Unbound proteins were removed by washing the beads five times in buffer B2 (30 mM HEPES, pH 7.6, 150 mM NaCl, 1 mM EDTA, 0.5% NP-40, and complete EDTA-free protease inhibitor cocktail) and centrifuged at 1000 rpm for 1 min at 4°C. Samples were eluted in Laemmli sample buffer by incubation for 5 min at 95°C and analyzed by immunoblot.

### Nascent transcription rates and recovery of RNA synthesis assays

Cells were grown on 24-mm glass coverslips in six-well plates 24 h before pre-treatment with PROTAC3 and DNA-damaging agents as indicated. Nascent transcription levels (nascent RNA) were measured by pulse labeling for 30 min at 37°C, 5% CO_2_ with the nucleotide analogue 5-ethynyluridine (100 mM) (EU, Jena Bioscience) in DMEM supplemented with 10% FBS and 1% PS. Cells were fixed in 3.6% formaldehyde (FA; Sigma) in PBS for 15 min and permeabilized with 0.1% Triton X-100 in PBS for 10 min at RT. Incorporated EU was fluorescently labeled via Click-it-chemistry-based azide coupling reaction by incubation with a cocktail containing 50 mM Tris buffer (pH 8), 60 μM Atto-Azide 594 nm (Atto-tech), 4 mM CuSO_4_·5H_2_O (Sigma), and 10 mM freshly prepared l-Ascorbic Acid (Sigma) for 1 h at RT in the dark. Thereafter, cells were washed 3× in 0.1% Triton X-100 in PBS for 5 min, washed 2× with PBS only, and then DNA was stained with 100 ng/ml of 4,6-Diamidino-2-phenylindole (DAPI; Sigma) in PBS for 15 min to visualize the nuclei. Finally, cells were mounted on glass slides (Epredia™ SuperFrost™) using Aqua-Poly/Mount (Polysciences) and air-dried o/n at RT in the dark.

Cells were imaged using a Zeiss LSM 700 Axio Imager Z2 upright microscope equipped with a 40× Plan-apochromat 1.3 NA oil-immersion lens (Carl Zeiss Micro Imaging) using Carl Zeiss LSM software (ZEN)(v.14.0.0.0). The integrated density of the EU signal in the nuclei was quantified using ImageJ/Fiji software (v.1.52p) with a macro to segment individual nuclei. The integrated density was normalized to the average integrated density of mock-treated cells, which was set at 100% and plotted as single-cell points.

### Immunofluorescence

Cells were grown on 24-mm glass coverslips at least 1 day before any treatment or fixation. Cells were treated with 100 μM cycloheximide 1 h prior to treatment and remained in the medium for the duration of Pol II degradation assays. Subsequently, cells were fixed in 3.6% FA for 15 min and permeabilized with 0.1% Triton X-100 in PBS for 10 min and blocked with PBS+ (0.15% BSA and 0.15% glycine in PBS) for 20 min. After washing in PBS, cells were incubated for 2 h at RT with proper primary antibodies diluted in 1% BSA in PBS. Thereafter, cells were washed 2× short and 2× extensively for 10 min in PBS TritonX-100 (0.5%). The secondary antibody and 100 ng/ml DAPI (Sigma) were also diluted in 1% BSA in PBS and incubated together on the cells for 1 h. After incubation, cells were washed again and then mounted on glass slides using Aqua-Poly/Mount (Polysciences).

Fluorescent images were acquired on a Zeiss LSM 700 Axio Imager Z2 upright microscope equipped with a 40× Plan-apochromat 1.3 NA oil-immersion lens (Carl Zeiss Micro Imaging) using ZEN software (v.14.0.0.0). Image analysis was performed in ImageJ/Fiji software (v.1.52p) with a macro segmenting the individual nuclei based on the DAPI signal. The integrated density was normalized to the average integrated density of mock-treated cells which was set at 100% and plotted as single-cell points.

### Live cell imaging and FRAP analysis

CSB and RPB1 fluorescence recovery after photobleaching (FRAP) experiments were performed as previously described [48, 68]. Cells were seeded on glass coverslips 2 days prior to the experiment and treated as indicated. In brief, for HCT116 CSB-mScarletI Halo-HA-INTS11 KI cells, imaging was performed using a Leica TCS SP8 confocal microscope (LAS AF software, Leica) equipped with an HC PL APO CS2 63× 1.40 NA oil immersion lens. Cells were placed in a controlled environment with 37°C and 5% CO_2_. FRAP analysis was performed by imaging 512 × 16 pixels across the nucleus of the cell. Fluorescence was measured at intervals of 400 ms for 5 frames pre-bleach, 2 frames of 50% laser power during photobleaching, and 30 frames post-bleach at 400 Hz speed using a 541-nm laser. For FRAP analysis in RPE1 GFP-RPB1 Halo-HA-INTS11 KI cells, imaging was performed using a Leica SP5 confocal microscope (LAS AF software, Leica) equipped with a HCX PL APO CS 63× 1.40 NA oil-immersion lens. Cells were placed in a controlled environment with 37°C and 5% CO_2_ during the experiments. Fluorescence of GFP-RPB1 was detected using a 488-nm argon laser. One-hundred percent laser power at 400 Hz for one frame was used to bleach a strip of 512 × 32 pixels across the nucleus of the cells. Fluorescence was measured at intervals of 400 ms for 25 frames pre-bleach and 600 frames post-bleach.

The fluorescence intensity of the nucleus in the bleached strip was background corrected to the pre-bleach fluorescence intensity outside of the nucleus within the same strip. Relative fluorescence intensity (RFI) was calculated by normalizing fluorescence values post-bleach to average values pre-bleach, which was set at 100%. Immobile fractions (*F*_imm_) were calculated by comparing the UV-irradiated conditions to the mock-treated condition for each cell line using the following formula:


\begin{eqnarray*}
\left( {{{F}_{\mathrm{ imm}}}\ = 1 - \ \frac{{\left( {{{I}_{\mathrm{recovery};\mathrm{ UV}}} - \ {{I}_{\mathrm{bleach};\mathrm{ UV}}}} \right)}}{{\left( {\left\langle {{{I}_{\mathrm{recovery};\mathrm{untreated}}}} \right\rangle \ - \ {{I}_{\mathrm{bleach};\mathrm{ UV}}}{\mathrm{\ }}} \right)}}} \right),
\end{eqnarray*}

where *I*_recovery;UV_ is the average RFI of frames 501–600 post-bleach (GFP-RPB1) or frames 26–30 post-bleach (CSB-mScarletI) of each UV-treated cell, *I*_bleach;UV_ is the RFI directly after bleaching, and < *I*_recovery;untreated_ > is the average RFI of frames 501–600 post-bleach (GFP-RPB1) or frames 26–30 post-bleach (CSB-mScarletI) of all cells in the untreated control cells. The same analysis was also done for FA-treated cells for Pol II FRAP with *I*_recovery;FA _instead of *I*_recovery;UV_ and *I*_bleach;FA_ instead of *I*_bleach;UV_.

### Alkaline comet assay

TC-NER-dependent trabectedin-induced single-strand breaks (SSBs) were quantified using the alkaline comet assay, as described previously [71]. Asynchronous HCT116 cells were pre-incubated with the DNA repair synthesis inhibitors hydroxyurea (HU, 1 mM; Sigma) and 1-β-d-arabinofuranosyl cytosine (AraC, 10 µM; Sigma) for 30 min at 37°C before treatment with trabectedin (30 nM; Sigma) for 2 h, without removing HU and AraC. Cells were collected by trypsinization, washed with PBS, and diluted in cold PBS to 3 × 10⁵ cells/ml. Cell suspensions were mixed with low-melting agarose (LMA; Trevigen) at a 1:10 (v/v) ratio, and 50 μl of the mixture was spread on CometSlides (Trevigen) to yield ∼1500 cells per well. After solidification at 4°C in the dark for 15 min, slides were immersed in pre-chilled lysis buffer (Trevigen) o/n at 4°C. DNA was unwound in freshly prepared alkaline solution (200 mM NaOH, 1 mM EDTA, pH > 13) for 1 h at RT in the dark, followed by electrophoresis at 1 V/cm (measured electrode to electrode) for 50 min at 4°C, in alkaline electrophoresis solution (200 mM NaOH, 1 mM EDTA, pH > 13) in the dark. Slides were washed twice in distilled water for 5 min, fixed in 70% ethanol for 5 min, and completely dried at 37°C in the dark. DNA was stained with SYBR Gold (1× in PBS; Invitrogen) for 30 min, rinsed in distilled water, and dried at 37°C. Comets were visualized using a Zeiss LSM 700 microscope at 10× magnification, and tail DNA percentage was quantified using CometScore software (TriTek; http://rexhoover.com/index.php?id=cometscore).

### Transcription-coupled unscheduled DNA synthesis

Transcription-coupled unscheduled DNA synthesis (TCR-UDS) was performed as previously described [72]. In brief, XPC-deficient RPE1 Halo-HA-INTS11 KI cells were grown on glass coverslips and arrested in G1/G0 with the CDK inhibitor palbociclib (1 µM; Selleck) for 24 h. Cells were labeled with 20 µM EdU (Sigma) and 1 µM floxuridine (Sigma) for 8 h at 37°C. After labeling, unincorporated EdU was removed by a chase in Ham’s F10 medium supplemented with 1 μM palbociclib, 10% FCS, and 1% penicillin/streptomycin with 10 μM thymidine (Sigma) for 15 min at 37°C. Cells were fixed with 3.6% FA and 0.5% Triton-X 100 in PBS for 15 min at RT and permeabilized with 0.5% Triton X-100 for 20 min followed by blocking in 3% BSA (Sigma). After blocking, cells were washed in PBS and the endogenous peroxidase activity was quenched by addition of 3% hydrogen peroxide (Sigma) in PBS for 30 min. Subsequently, cells were washed in PBS with 0.5% BSA and 0.15% glycine (PBS+) for 1 h. A click-it-chemistry-based reaction was performed by addition of a cocktail containing 1× Click-it reaction buffer (Thermo Fisher Scientific), copper(III) sulfate (4 mM), azide–PEG3–biotin conjugate (20 µM, Jena Bioscience), and 10× reaction buffer additive (Thermo Fisher Scientific) for 30–60 min and washed three times with PBS. Thereafter, the UDS signal was amplified using HRP–streptavidin conjugate (500 µg/ml) (Thermo Fisher, B40932) for 1 h at RT. Cells were washed three times with PBS and Alexa-Fluor-488-labeled tyramide (100× stock, Thermo Fisher Scientific) was added for 10 min. Lastly, a “reaction stop” reagent working solution was added to the cells for 2–3 min and after washing, cells nuclei were stained using DAPI (100 ng/ml in PBS, Brunschwig Chemie). Finally, coverslips were mounted with Aqua-Poly/Mount (Polysciences) to microscope slides (Epredia SuperFrost) and air-dried o/n at RT and stored at 4°C. Fluorescent images were captured using a Zeiss LSM 700 Axio Imager Z2 upright microscope equipped with a 40× Plan-apochromat 1.3 NA oil-immersion lens (CarlZeiss Micro Imaging). The EdU signal in the nuclei was quantified using ImageJ/Fiji.

### Flow cytometry

Cells were grown in six-well plates 2 days before treatment and flow cytometry analysis. When indicated, cycloheximide (100 μM, Sigma) was added to the cells 1 h prior to DNA damage induction and kept on the cells for the duration of the experiment to prevent *de novo* protein synthesis. Cells were collected by trypsinization, centrifuged for 3 min at 281 × *g*, and resuspended in 500 μl PBS containing 1% FA at the indicated timepoints. Samples were kept on ice in the dark until analysis.

Fluorescence levels were analyzed on an LSRFortessa Cell Analyzer (BD) equipped with FACSDiva Software (BD) and >10 000 cells were recorded per experiment. After exclusion of apoptotic/dead cells by gating for live cells based on granularity (SSC-A) and size (FSC-A), and doublet exclusion based on FSC-H versus FSC-W and SSC-H versus SSC-W gating, GFP-RPB1 fluorescence levels were detected using a 488-nm laser in combination with a 530/30 filter. Flow cytometry data were analyzed using FlowJo software (v.10.8.1) from BD Biosciences and used to determine the mean fluorescence intensity of GFP-RPB1. Fluorescence intensity was background-corrected using non-fluorescent cells and normalized to mock-treated fluorescence, which was set to 100.

### Local DRB run-off immunofluorescence microscopy assay

The local DRB run-off assay was used to study chromatin binding of lesion-stalled Pol II as described previously [29, 73]. Cells were seeded on coverslips a day prior to treatment. Local UV damage was induced by irradiating cells through 5-μm pore polycarbonate filters (Millipore) with 80 J/m² UV-C. Directly after damage induction, cells were treated with 100 μM DRB (Sigma) for the indicated times, followed by PBS washes and fixation with 3.6% FA in PBS for 15 min at RT. Fixed cells were permeabilized with 0.5% Triton X-100 in PBS for 10 min, and for CPD detection, DNA was denatured with 0.07 M NaOH for 5 min at RT. After blocking with 100 mM glycine in PBS and subsequently with 0.5% BSA/0.05% Tween-20 in PBS, samples were incubated for 2 h at RT with antibodies against pSer-Pol II (ab5095, Abcam; 1:1000) and CPDs (CAC-NM-DND-001, Cosmo Bio; 1:1000). After washes, Alexa Fluor 488-conjugated anti-mouse (A-11029, Thermo Fisher) and Alexa Fluor 555-conjugated anti-rabbit (A-21429, Thermo Fisher) secondary antibodies, together with DAPI, were applied for 1 h at RT. Coverslips were mounted on glass slides using Aqua-Poly/Mount (Polysciences). Fluorescent images were acquired on a Zeiss LSM 700 Axio Imager Z2 upright microscope equipped with a 40× Plan-apochromat 1.3 NA oil-immersion lens (Carl Zeiss Micro Imaging) using ZEN software (v.14.0.0.0). Image analysis was performed in ImageJ/Fiji software (v.1.52p) with a macro segmenting nuclei based on the DAPI signal. Sites of local damage was detected by CPD staining, after which the ratio of the pSer2-Pol II inside damage (at sites of local damage) compared to outside of the damage (non-damaged areas of the nucleus) was plotted per cell with local damage.

### Statistical analysis

Experimental data were analyzed and processed in Excel and plotted using Graph Pad Prism (version 10.3.1) (GraphPad Software). The number of experiments and statistic tests (unpaired two-tailed *t*-tests) can be found in the figure legends. *P*-values expressed as *P < .05; **P < .01, ***P < .001, and **** *P < *.0001 were considered to be significant. ns, non-significant.

## Results

### Integrator complex interacts with elongating Pol II and the TC-NER machinery

A powerful approach to identify factors involved in TC-NER is to compare the composition of the Pol II–TC-NER complex in wild-type (WT) cells with that in TC-NER-deficient cells. In the deficient cells, repair intermediates will accumulate at a defined stage of the repair reaction, preventing the recruitment of downstream factors. To identify factors involved in Pol II processing, we compared the lesion-stalled Pol II complex in TC-NER proficient cells with that of STK19 knockout (KO) cells, since STK19 is required for CRL4^CSA^ activity and proper TFIIH integration, and its loss therefore traps the repair reaction at one of the final steps before stalled Pol II must be processed to allow excision of the damage [27, 29, 30, 32]. To do so, we analyzed SILAC-based interaction proteomics data of transcriptionally engaged Pol II (pSer2-modified) interactomes in WT and STK19 KO cells upon UV exposure [27]. As previously described, binding of the TC-NER factors UVSSA and TFIIH was severely impaired in STK19 KO cells (Fig. 1A and B). Interestingly, gene ontology (GO) analysis revealed that, alongside these TC-NER factors, the Integrator complex was the top factor with reduced Pol II binding in STK19 KO cells (Fig. 1C and Supplementary Fig. S1A and B). Binding of all quantified Integrator complex subunits (13 out of 15) to transcriptionally engaged Pol II in response to UV damage was reduced to a similar extent as that of UVSSA and TFIIH subunits. This indicates that the Integrator complex binds to the Pol II-TC-NER complex depending on the presence of STK19, and suggests a possible role for this multi-subunit regulator of Pol II-mediated transcription and RNA processing in TC-NER [55, 56]. In line with this, previous proteomics and siRNA screens have suggested a putative role for the Integrator complex in the cellular response to transcription-blocking damage [52].

**Figure 1.**
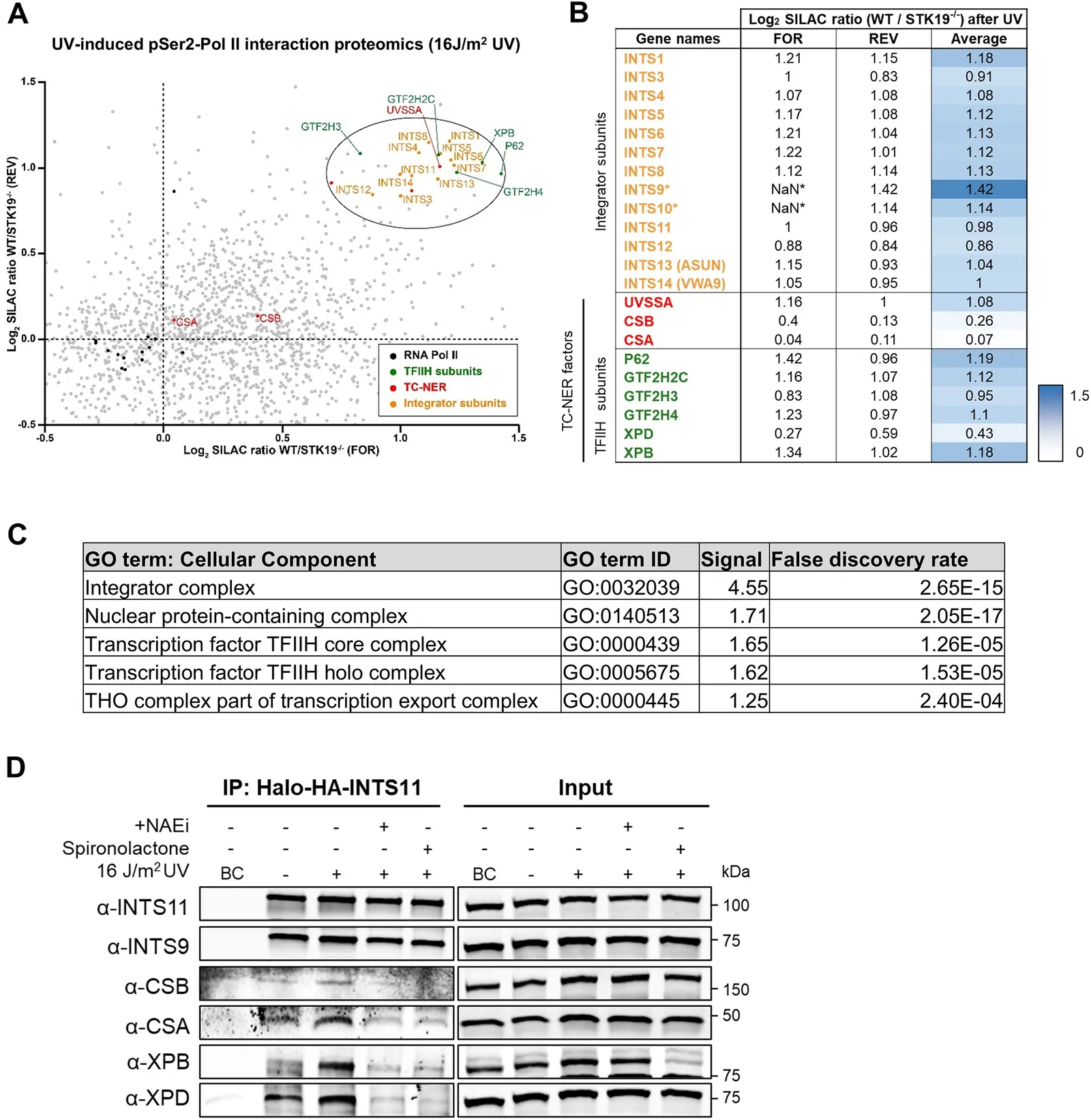
Integrator complex binds to lesion-stalled Pol II dependent on STK19. (**A**) Reanalysis of SILAC-based quantitative interaction proteomics of the UV-induced pSer2-modified RPB1 subunit of Pol II. Log2 SILAC ratios of HCT116 WT versus STK19 knockout (KO) cells upon UV irradiation (16 J/m^2^, 1 h), including a SILAC label-swap replicate, were plotted. Pol II subunits are depicted in black. TC-NER proteins are indicated in red, subunits of the TFIIH complex in green, and subunits of the Integrator complex in orange. (**B**) Log2 SILAC ratios of pSer2-modified Pol II interactors in WT versus STK19 KO cells upon UV-damage as shown in panel (A). (**C**) Top 5 enriched GO terms (cellular component) identified using g:Profiler of STK19-dependent proteins that bind to elongating Pol II upon UV-induced damage as shown in Fig. 1A, with a Log2 SILAC ratio >0.6. (**D**) IP in the solubilized chromatin fraction of Halo-HA-INTS11 using HA beads in Halo-HA-INTS11 KI cells followed by immunoblotting for the indicated proteins. Where indicated, cells were pretreated with NAEi (10 µM, 30 min) or spironolactone (10 µM, 2 h) before harvesting. Cells were collected 1 h after mock treatment or after irradiation with 16 J/m^2^ UV. BC: binding control.

To study the role of the Integrator complex during TC-NER, we generated a homozygous Halo-HA-tagged INTS11 knock in (Halo-HA-INTS11 KI) cell line by CRISPR-mediated gene editing (Supplementary Fig. S1C–F). Halo-HA-INTS11 was expressed at endogenous levels, with a normal nuclear localization (Supplementary Fig. S1G and H). Furthermore, nascent transcription rates, measured by 5-ethynyl uridine (EU) incorporation [74], were comparable to those in WT cells, indicating that Halo-HA-INTS11 did not perturb endogenous transcription (Supplementary Fig. S1I). This cell line enables efficient detection, IP, and rapid depletion of the catalytic Integrator subunit INTS11 [57, 58, 63].

To confirm that the Integrator complex was part of the TC-NER complex, we immunoprecipitated INTS11 from the benzonase-solubilized chromatin fraction, as TC-NER complexes are tightly chromatin bound [17, 25]. As expected, INTS11 interacted with INTS9, which, together with INTS4, forms the catalytic core of the Integrator complex [57–59], irrespective of DNA damage induction (Fig. 1D). Following UV-induced DNA damage, INTS11 additionally interacted with CSB, CSA, and TFIIH subunits XPB and XPD, indicating association of the Integrator complex with the TC-NER complex. The UV-induced interaction of INTS11 with the TC-NER complex was lost upon the treatment with NEDD8-activating enzyme inhibitor (NAEi), which inhibits CRL4^CSA^ and thereby UV-induced Pol II ubiquitylation [22, 75]. Similarly, treatment with spironolactone, which degrades XPB and prevents TFIIH from being recruited to the TC-NER complex [76], disrupted the INTS11 binding to these TC-NER factors. Together, these findings demonstrate that the Integrator complex is recruited to late-stage TC-NER complexes downstream of CRL4^CSA^-mediated Pol II ubiquitylation and TFIIH recruitment.

### INTS11 promotes TC-NER-mediated transcription recovery

Transcription recovery after DNA damage is a key hallmark for TC-NER [77]. Therefore, we tested transcription recovery after UV irradiation, by quantifying nascent transcription levels by EU incorporation [74], in the presence or absence of INTS11. To achieve rapid depletion of INTS11, we employed HaloPROTAC3, a proteolysis-targeting chimera (PROTAC) that directs the E3 ubiquitin ligase VHL to Halo-HA-tagged INTS11 [78, 79]. INTS11 was almost completely depleted 6 h after PROTAC3 treatment (Fig. 2A). INTS11 depletion also reduced INTS9 levels, but had no significant effect on Pol II protein levels (Fig. 2B and Supplementary Fig. S2A). Moreover, INTS11 depletion slightly increased transcription rates under unperturbed conditions (Supplementary Fig. S2B), likely as loss of Integrator reduces both promoter-proximal pausing and premature transcription termination and results in upregulation of short RNA transcripts [63]. Transcription was severely impeded 1 h after UV treatment in all cell lines (Fig. 2C and Supplementary Fig. S2C). Interestingly, while transcription levels fully recovered after 16 h in the presence of INTS11, PROTAC3-mediated INTS11 depletion severely inhibited transcription recovery, to almost a similar extent as observed in TC-NER-deficient CSB-depleted cells. Similar results were obtained in RPE1 Halo-HA-INTS11 KI cells upon PROTAC3-mediated INTS11 depletion (Supplementary Fig. S2D and E).

**Figure 2.**
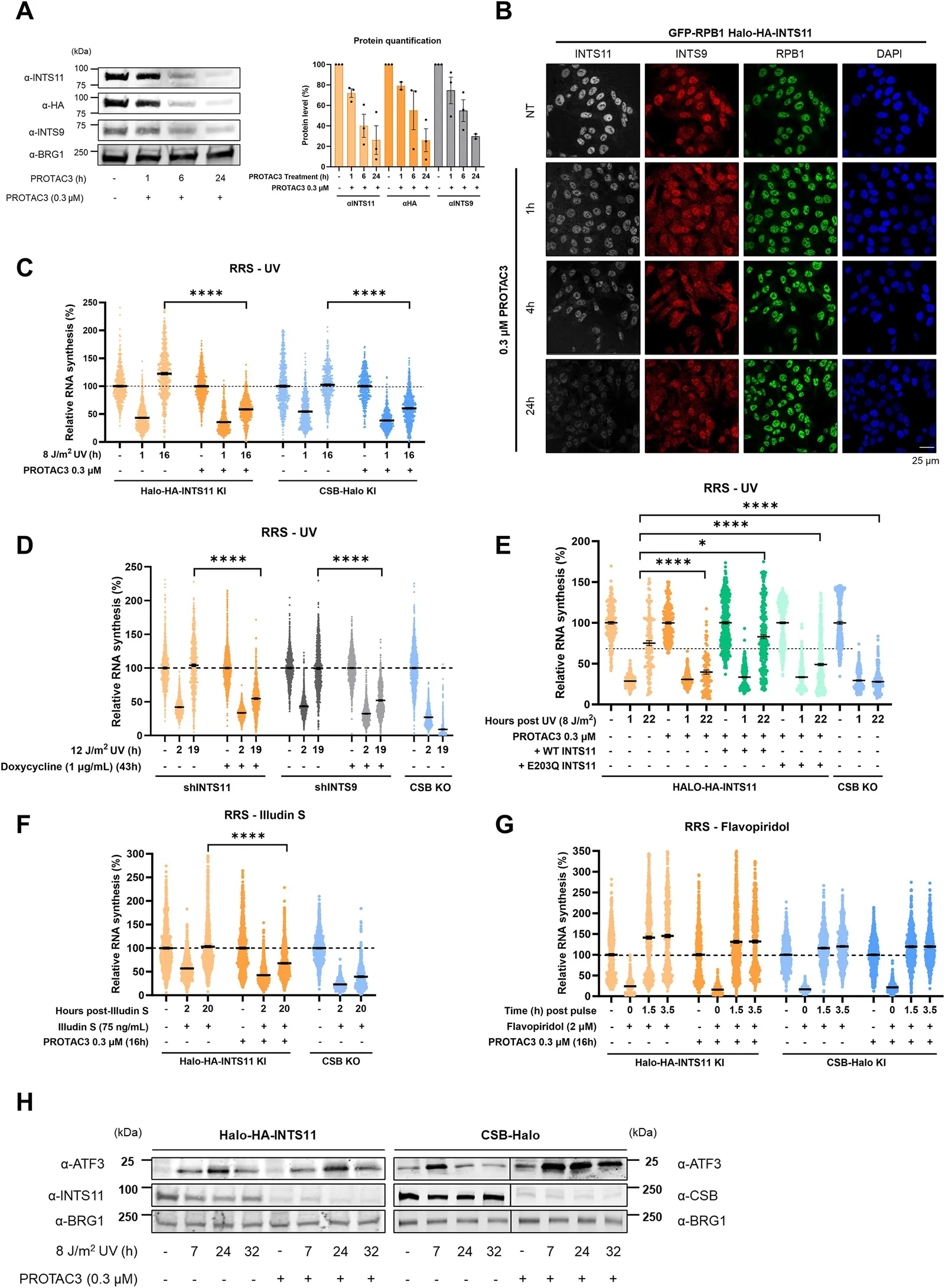
Integrator complex facilitates transcription recovery after transcription-blocking damage. (**A**) Immunoblot analysis (left) and quantification (right) of INTS11, INTS9, and HA from lysates of HCT116 Halo-HA-INTS11 KI cells treated with PROTAC3 (0.3 µM) for the indicated timepoints. Protein levels were normalized to the loading control. BRG1 was used as a loading control and set at 100%. This experiment was performed three times with similar results. (**B**) Representative images of INTS11, INTS9, and RPB1 as determined by IF for INTS11 and INT9, and GFP signal for RPB1 in non-treated or 1, 4, and 24h after PROTAC3 (0.3 µM) treatment. NT, non-treated. Scale bar, 25 µm. (**C**) Quantification of nascent transcription levels as determined by EU incorporation (30 min) at the indicated timepoints after UV irradiation (8 J/m^2^) in HCT116 Halo-HA-INTS11 KI cells and HCT116 CSB-Halo cells. Where specified, cells were pre-treated with PROTAC3 (0.3 µM, 16 h) to deplete INTS11 or CSB. Mean RFI of EU (relative RNA synthesis) were normalized to untreated levels and set to 100% (dotted line). Black lines indicate average integrated density ± SEM from three independent experiments (*n* ≥ 546 cells per condition). Statistical significance was determined using an unpaired two-tailed Student’s *t*-test (****P < .0001). (**D**) Quantification of nascent transcription levels as determined by EU incorporation (30 min) at the indicated timepoints after UV irradiation (12 J/m^2^) in HeLa cells in which short hairpin RNA (shRNA) targeting INTS11 and INTS9 were induced by doxycycline (1 µg/ml). Mean RFI of EU (relative RNA synthesis) were normalized to untreated levels and set to 100% (dotted line). Black lines indicate average integrated density ± SEM from three independent experiments (at least *n* ≥ 662 cells per condition). Statistical significance was determined using an unpaired two-tailed Student’s *t*-test (****P < .0001). (**E**) Quantification of nascent transcription levels as determined by EU incorporation (30 min) at the indicated timepoints after UV irradiation (8 J/m^2^) in RPE1 Halo-HA-INTS11 KI cells in which either WT or catalytic mutant (E203Q) was lentivirally expressed as indicated, and RPE1 CSB KO cells. Where specified, cells were pre-treated with PROTAC3 (0.3 µM, 16 h) to deplete endogenous INTS11. Mean RFI of EU (relative RNA synthesis) were normalized to untreated levels and set to 100% (dotted line). Black lines indicate average integrated density ± SEM from at least three independent experiments (*n* ≥ 93 cells per condition). Statistical significance was determined using an unpaired two-tailed Student’s *t*-test (*P < .05, ****P < .0001). (**F**) Quantification of nascent transcription levels as determined by EU incorporation (30 min) at the indicated timepoints after Illudin S-induced damage (75 ng/ml for 2.5 h) in HCT116 Halo-HA-INTS11 KI cells and HCT116 CSB KO cells. Illudin S was washed away with PBS and transcription was assessed at the indicated times after Illudin S washout. Where specified, cells were pre-treated with PROTAC3 (0.3 µM, 16 h) to deplete INTS11. Mean RFI of EU (relative RNA synthesis) were normalized to untreated levels and set to 100% (dotted line). Black lines indicate average integrated density ± SEM from three independent experiments (*n* ≥ 590 cells per condition). Statistical significance was determined using an unpaired two-tailed Student’s *t*-test (****P < .0001). (**G**) Quantification of nascent transcription levels as determined by EU incorporation (30 min) at the indicated timepoints after flavopiridol washout in HCT116 Halo-HA-INTS11 KI and HCT116 CSB-Halo cells. Cells were treated with flavopiridol (2 µM) for 2.5 h and then flavopiridol was washed out and transcription was assessed at the indicated times after washout. Where specified, cells were pre-treated with PROTAC3 (0.3 µM, 16 h) to deplete INTS11 or CSB. Mean RFI of EU (relative RNA synthesis) were normalized to untreated levels and set to 100% (dotted line). Black lines indicate average integrated density ± SEM from three independent experiments (*n* ≥ 558 cells per condition). “−” = non-treated, “0h” = EU incorporation during flavopiridol treatment. (**H**) Immunoblot analysis for the indicated antibodies of ATF3 signal of lysates of RPE1 Halo-HA-INTS11 KI and RPE1 CSB-Halo KI cells before or 7, 24, or 32 h after UV treatment (8 J/m^2^). Where indicated, cells were pre-treated with PROTAC3 (0.3 µM, 16 h). BRG1 was used as a loading control. This experiment was performed three times with similar results. Several western blot images were cropped for presentation. For RPE1 CSB-Halo KI cells, all lanes shown originate from the same membrane and exposure.

To test whether this transcription recovery defect is specific for INTS11 depletion or reflects a broader requirement of the Integrator complex, we depleted also the INTS9 subunit of the catalytic endonuclease core of the Integrator complex. Short hairpin RNA (shRNA)-mediated INTS9 depletion showed a similar impairment in transcription recovery as observed upon INTS11 depletion [55, 64] (Fig. 2D). To determine whether the endonuclease activity of the Integrator complex is required for transcription recovery following TBL induction, we tested a catalytic inactive INTS11 [55, 64, 69] (Fig. 2E and Supplementary Fig. S2F). Re-expression of the E203Q catalytic inactive INTS11 mutant in INTS11-depleted cells failed to restore transcription, resulting in transcription levels comparable to those observed in INTS11-depleted cells. In contrast, re-expression of WT INTS11 successfully rescued transcription recovery. Together, these results indicate that the endonuclease activity of the Integrator complex stimulates transcription recovery upon UV-induced damage.

To determine whether INTS11 depletion also impairs transcription recovery following other types of TBLs, we examined the effect of Illudin S, which induces lesions that are repaired specifically by TC-NER and not by GG-NER [80]. Exposure to Illudin S led to a comparable level of inhibition as that of UV, and in WT cells transcription fully recovered 20 h after exposure (Fig. 2F and Supplementary Fig. S2G). Similar to UV-induced damage, loss of INTS11 resulted in reduced transcription recovery after Illudin S treatment. Of note, the recovery in INTS11-depleted cells was not as severely impeded as in CSB KO cells, possibly caused by residual INTS11 levels due to incomplete PROTAC3-mediated INTS11 degradation.

Given the broad function of the Integrator complex during transcription [81], we tested whether the observed transcription recovery after TBLs could be caused by a general defect in transcription initiation. Therefore, we tested the effect of INTS11 depletion on transcription recovery upon transcription inhibition with the reversible CDK9 inhibitor flavopiridol, which inhibits Pol II release from the promoter-proximal pause site [82]. As expected, flavopiridol treatment severly inhibited transcription (Fig. 2G) and flavopiridol washout restored nascent transcription levels through *de novo* initiation in WT cells [83]. Similar recovery levels were observed in WT and in CSB- and INTS11-depleted cells, demonstrating that the Integrator complex is specifically required for transcription recovery upon transcription-blocking DNA damage.

TBLs induce expression of the transcriptional repressor ATF3, which contributes to damage-induced transcriptional shutdown [84–86]. Consistent with these findings, we observed that ATF3 protein levels were upregulated 7 h after UV irradiation (Fig. 2H). Importantly, it was shown that in the absence of functional CSA, CSB [84–86], ELOF1 [26], or STK19 [29], ATF3 levels remain elevated at timepoints when transcription recovers in TC-NER proficient cells (Supplementary Fig. S2H). These findings highlight that prolonged ATF3 accumulation serves as a robust marker of persistent transcription stress caused by defective TC-NER. In line with this, ATF3 remained significantly elevated at later timepoints in CSB-depleted cells, consistent with persistent transcription stress in these cells (Fig. 2H). In contrast, in INTS11-depleted cells ATF3 levels declined after 32 h, closely resembling the kinetics observed in TC-NER proficient WT cells. These results suggest that, unlike TC-NER–deficient cells, Integrator-deficient cells do not sustain prolonged ATF3 accumulation, indicating that the transcription recovery defect in this context is regulated through a distinct mechanism.

### DNA repair by TC-NER is independent of INTS11

The reduced transcription recovery observed in INTS11-depleted cells may arise from defective TC-NER or INTS11 may have a role downstream of TC-NER, specifically facilitating transcription recovery following transcription-blocking damage. Therefore, we studied whether INTS11 is required for TC-NER-mediated repair. TC-NER is initiated by the detection of lesion-stalled Pol II by CSB. The CSB interaction with lesion-stalled Pol II was studied by FRAP using CSB-mScarletI KI cells, which is a sensitive tool to study binding of CSB to lesion-stalled Pol II [12, 68]. CSB-mScarletI FRAP revealed a strong CSB immobilization upon UV exposure in INTS11-depleted cells, comparable to WT cells (Fig. 3A), indicating that CSB is correctly recruited to lesion-stalled Pol II in the absence of INTS11. In WT cells, CSB remobilized almost completely 4 h after UV induction (Fig. 3A), consistent with TC-NER mediated repair of TBLs [25, 27, 68]. A similar remobilization was observed in INTS11-depleted cells, suggesting that INTS11 is not essential for TC-NER. Of note, CSB levels were not affected by INTS11 depletion (Supplementary Fig. S3A).

**Figure 3.**
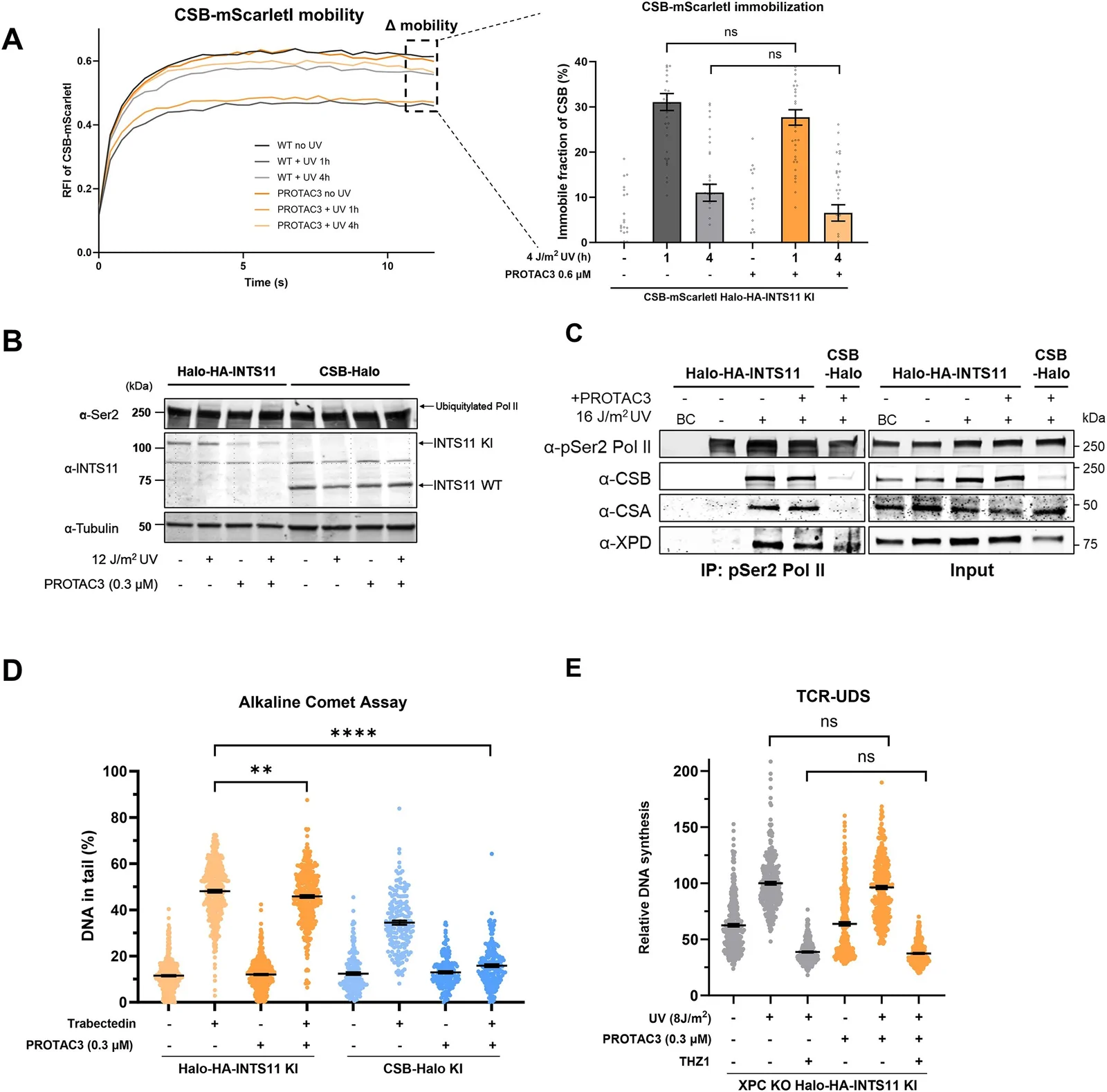
INTS11 is not essential for TC-NER. (**A**) (Left) FRAP analysis of CSB in HCT116 CSB-mScarletI Halo-HA-INTS11 KI cells upon UV irradiation (4 J/m^2^). Cells were pretreated with PROTAC3 (0.6 µM, 24 h) as indicated. CSB-mScarletI was bleached in a strip across the nucleus and fluorescence intensity was measured every 0.4 s for 11.6 s in untreated cells or at the different timepoints after UV irradiation (4 J/m^2^). *n* = 36 cells per condition from at least three independent experiments. CSB-mScarletI RFI was background-corrected and normalized to pre-bleach fluorescence intensity and set to 1. (Right) Calculated immobile fraction of CSB-mScarletI after DNA damage obtained from the data points in the dotted box indicated in left (timepoints 10.0 s–11.6 s). Immobile fraction in WT and INTS11-depleted cells was calculated by comparing RFI after UV to mock-treated sample and normalizing to their own non-irradiated RFI values. *n* = 36 cells per condition from three independent experiments ± SEM. Statistical significance was determined using an unpaired two-tailed Student’s *t*-test (ns, non-significant). (**B**) Immunoblot using the indicated antibodies of the chromatin fraction of HCT116 Halo-HA-INTS11 or CSB-Halo KI cells 1 h after 12 J/m^2^ UV or mock treatment. Where specified, cells were pre-treated with PROTAC3 (0.3 µM, 16 h) to deplete INTS11 or CSB. Tubulin was used as a loading control. (**C**) IP in the solubilized chromatin fraction of pSer2 Pol II in mock- and UV-treated (16 J/m^2^, 1 h) HCT116 CSB-mScarletI and Halo-HA-INTS11 KI cells and CSB-Halo KI cells, followed by immunoblot with the indicated antibodies. Where indicated, cells were pre-treated with PROTAC3 (0.6 µM, 24 h) to deplete INTS11. Right, chromatin-fraction input samples. BC, binding control agarose beads. (**D**) Alkaline comet assays of HCT116 Halo-HA-INTS11 KI cells and HCT116 CSB-Halo KI cells upon pre-treatment with PROTAC3 (0.6 µM, 24 h) as indicated, either untreated or exposed to trabectedin (30 nM) for 2 h in the presence of HU (1 mM) and AraC (10 µM). The percentage of DNA present in the comet tail (%) per cell was plotted. Black lines indicate average integrated density of the DNA in the tail ± SEM from *n* = 6 for Halo-HA-INTS11 KI cells and *n* = 3 for CSB-Halo KI cells (*n* ≥ 169 cells per condition). Statistical significance was determined using an unpaired two-tailed Student’s *t* (**P < .01, ****P < .0001). (**E**) TCR-UDS as determined by EdU incorporation upon UV exposure (8 J/m^2^) in RPE1 XPC KO Halo-HA-INTS11 KI cells. Where specified, cells were pre-treated with PROTAC3 (0.3 µM, 24 h) and/or with 2 μM THZ1. Mean RFI of fluorescent labeled EdU was normalized to “+UV −PROTAC3” levels and set to 100%. Black lines indicate average integrated density ± SEM from three independent experiments (*n* ≥ 302 cells per condition). Statistical significance was determined using an unpaired two-tailed Student’s *t*-test (ns, non-significant).

Pol II ubiquitylation by CRL4^CSA^ is a critical reaction step during TC-NER, as it stimulates TC-NER complex assembly by promoting UVSSA and TFIIH recruitment [22, 25, 26]. Depletion of INTS11 had no effect on UV-induced Pol II ubiquitylation (Fig. 3B), indicating that CRL4^CSA^ activity was not impeded in the absence of INTS11. Furthermore, Pol II ubiquitylation declined with similar kinetics at later timepoints in both WT and INTS11-depleted cells, consistent with normal progression of TC-NER (Supplementary Fig. S3B). The normal induction of Pol II ubiquitylation indicates that the conditions required for proper UVSSA and TFIIH recruitment remained intact. To confirm this, we assessed TFIIH recruitment to lesion-stalled Pol II in the absence of INTS11. To do so, we immunoprecipitated elongating Pol II from chromatin after UV irradiation [17, 25]. Both in WT and in INTS11-depleted cells, the UV-induced recruitment of CSB, CSA, and of the XPD subunit of the TFIIH complex to elongation Pol II was unaffected (Fig. 3C), whereas these interactions were, as expected, abolished in CSB-depleted cells [17, 27]. Collectively, these results show that the TC-NER complex assembly up to the stage of TFIIH recruitment remains unaffected in the absence of INTS11.

TFIIH, together with XPA and RPA, recruits the endonucleases XPG and ERCC1-XPF to incise and remove the TBL [87]. We therefore examined whether INTS11 affected the more downstream incision step of TC-NER. To test this, we made use of DNA adducts induced by exposure to trabectedin (ET-743), which are recognized and processed by TC-NER. However, as the bulky trabectedin adduct prevents XPG incision, only ERCC1-XPF introduces an incision, creating a persistent ssDNA breaks [88–90]. This property was utilized to examine the involvement of INTS11 in TC-NER-mediated TBL incision. As expected, in WT cells, using the alkaline comet assay, a strong increase of ssDNA breaks in response to trabectedin was observed (Fig. 3D), which was absent in CSB-depleted cells [90]. Interestingly, INTS11-depleted cells showed no decrease in trabectedin-induced ssDNA breaks, indicating that XPG and ERCC1-XPF are recruited normally and the TC-NER-mediated TBL incision still occurs in the absence of INTS11.

Following incision and TBL eviction, the resulting ssDNA gap is filled by repair synthesis to enable transcription recovery [2]. To determine whether INTS11 contributes to TC-NER-mediated gap filling, we quantified TCR-UDS using 5-ethynyl-2′-deoxyuridine (EdU) incorporation in non-cycling GG-NER-deficient XPC KO cells, allowing specific assessment of TC-NER-dependent repair [72]. In INTS11 proficient cells, a clear UV-induced TCR-UDS signal was observed, which was fully dependent on transcription, as blocking *de novo* transcription initiation using the CDK7 inhibitor THZ1 [91] completely inhibited TCR-UDS (Fig. 3E and Supplementary Fig. S3C). INTS11-depleted cells showed similar TCR-UDS signal upon UV as control cells, indicating that TC-NER-mediated gap filling proceeds normally in the absence of INTS11. Collectively, these findings demonstrate that INTS11 is dispensable for TC-NER-mediated lesion repair and instead functions downstream of TC-NER to promote transcription recovery.

### INTS11 facilitates Pol II processing at TBLs

As INTS11 is dispensable for TC-NER-mediated repair of TBLs, the defect in transcription recovery might result from prolonged retention of Pol II after repair, which could obstruct transcription by newly initiated Pol II. Given that the endonuclease activity of INTS11 promotes RNA cleavage to remove stalled or excessively paused Pol II, we investigated whether INTS11 also promotes the removal of lesion-stalled Pol II. To investigate this, we first evaluated Pol II degradation by measuring endogenous GFP-RPB1 protein levels using flow cytometry, which is a sensitive approach to quantify degradation of cellular Pol II [50, 67]. We assessed this in the presence of the protein synthesis inhibitor cycloheximide to exclude contributions from *de novo* protein translation [50]. Consistent with previous observations [27, 48, 50], upon UV damage, total Pol II levels gradually dropped in WT cells (Fig. 4A). This Pol II degradation was completely lost upon addition of NAEi, which prevents the activation of cullin-based ubiquitin ligases [75], confirming that the loss of Pol II reflects ubiquitin-dependent degradation. Notably, this Pol II degradation mainly represents degradation of non-lesion stalled Pol II in a genome-wide manner, which happens in a CSB and CRL4^CSA^-independent manner [48]. In INTS11-depleted cells, a comparable Pol II degradation as in WT cells was observed, indicating that INTS11 does not have a role in Pol II degradation, in line with our findings that INTS11 depletion does not affect Pol II ubiquitylation (Fig. 3B and Supplementary Fig. S3B). Similar results were also obtained in RPE1 cells (Supplementary Fig. S4A). This flow cytometry-based Pol II quantification approach determines the total Pol II levels in the cell. To more specifically focus on the chromatin-bound Pol II levels, we performed IF, which partially releases non-DNA bound Pol II [48, 67]. Consistent with the flow cytometric Pol II quantification, IF-based total Pol II levels were reduced upon UV, however this happens to a lesser extent upon INTS11 depletion (Fig. 4B). Notably, while the pSer2-modified transcriptionally engaged Pol II fraction decreased by ∼50% in WT cells, these Pol II levels remained largely unchanged in INTS11-depleted cells (Fig. 4B). Together, these findings suggest that the total levels of Pol II are not differentially affected upon DNA damage in INTS11-depleted cells, but that a larger fraction of pSer2-modified Pol II remains chromatin bound upon DNA damage.

**Figure 4.**
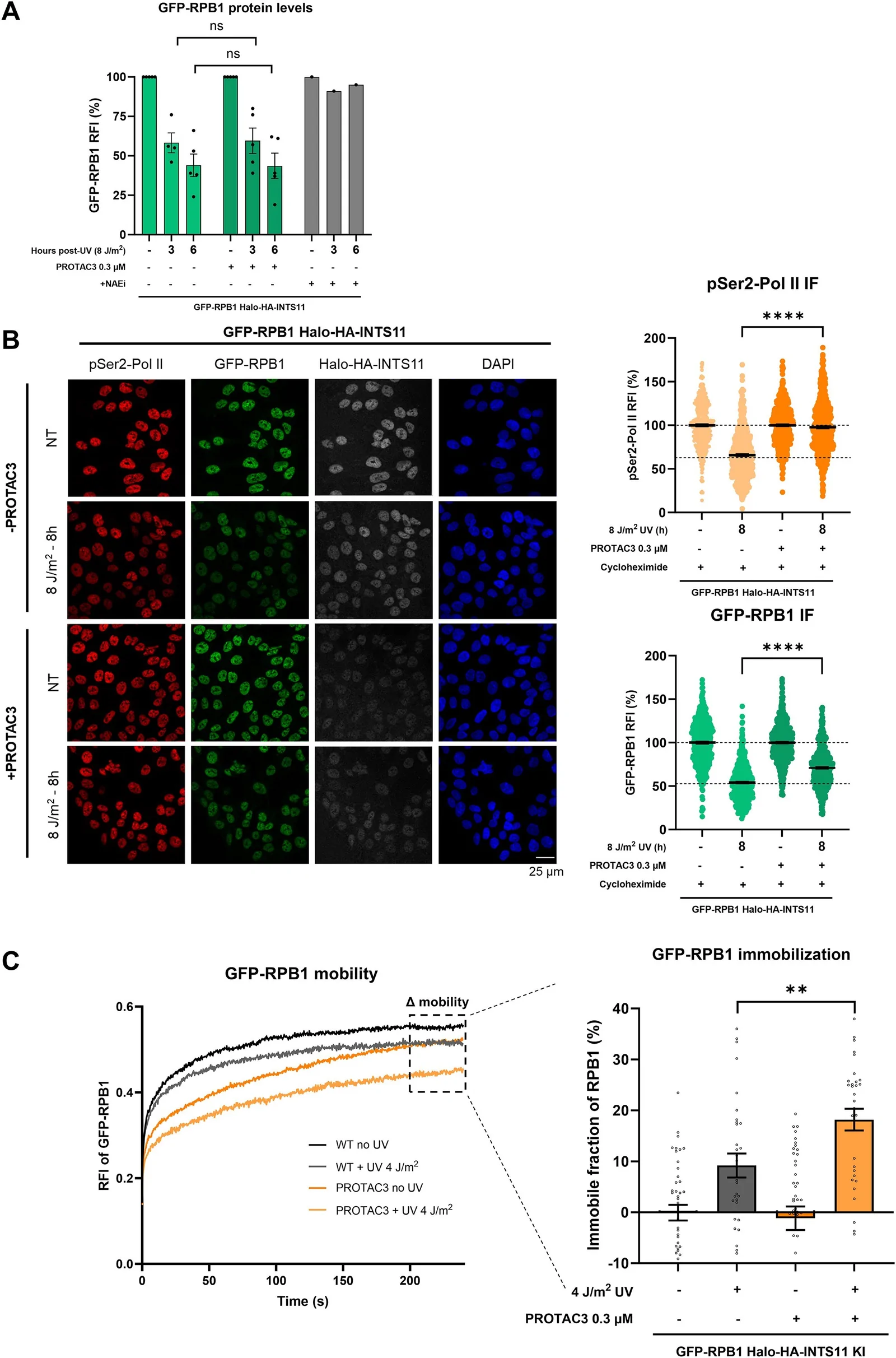
INTS11 results in prolonged chromatin-binding of Pol II after DNA damage. (**A**) Pol II levels in HCT116 GFP-RPB1 Halo-HA-INTS11 KI cells were quantified by flow cytometry. Where indicated, cells were pre-treated with PROTAC3 (0.3 µM, 16 h) and/or NAEi (10 μM, 30 min) to block CRL4^CSA^ activity. Cells were mock-treated or irradiated with UV (12 J/m^2^) and analyzed at the indicated timepoints. GFP-RPB1 levels in UV-irradiated cells were normalized to undamaged controls for each cell line (set at 100). Columns represent the mean RFI of at least four independent experiments of >10 000 cells ± SEM. Statistical significance was assessed by an unpaired two-tailed Student’s *t*-test (ns, non-significant). (**B**) (Left) Representative IF images of pSer2-modified Pol II, GFP-RBP1, and INTS11 levels in GFP-RPB1 Halo-HA-INTS11 KI cells either non treated (NT) or 8 h after UV exposure (8 J/m^2^). Cycloheximide (100 μM) was added 2 h prior to UV-irradiation and maintained for the duration of the experiment. Where indicated, cells were pre-treated with PROTAC3 (0.3 µM, 16 h). Scale bar, 25 µm. (Right) Quantification of elongating pSer2-modified Pol II- (top chart) and total Poll II levels (bottom chart) as determined by GFP-RPB1 levels as determined by IF. The RFI was normalized to mock-treated levels and set at 100. Black lines indicate average RFI intensity and error bars ± SEM. *n* ≥ 418 cells per condition from three independent experiments. Statistical significance was determined using an unpaired two-tailed Student’s *t*-test (****P < .0001). (**C**) (Left) FRAP analysis of Pol II mobility in mock- and UV-treated (4 J/m^2^, 1 h) RPE1 GFP-RPB1 Halo-HA-INTS11 KI cells. PROTAC3 (0.3 µM, 24 h) pre-treatment was done when indicated. GFP-RPB1 RFI was background-corrected and normalized to pre-bleach fluorescence intensity and set to 1. *n* ≥ 31 cells per condition from at least four independent experiments. (Right) Calculated immobile fraction of GFP-RPB1 after DNA damage obtained from the data points in the dotted box indicated in left (timepoints 199.4 s–239.4 s). Immobile fraction in WT and INTS11-depleted cells was calculated by comparing RFI after UV to mock-treated sample and normalizing to their own non-irradiated RFI values. *n* ≥ 31 cells per condition from at least four independent experiments ± SEM. Statistical significance was determined using an unpaired two-tailed Student’s *t*-test (**P < .01).

To test this, we measured Pol II chromatin binding dynamics using FRAP of GFP-tagged Pol II using GFP-RPB1 KI cells [67]. Pol II FRAP is a sensitive tool to study the Pol II dynamics in living cells, at different stages of the transcription cycle under unperturbed conditions [67] and to quantify DNA damage-induced perturbations of elongating Pol II [12, 25, 48]. The multifaceted role of the Integrator complex throughout different stages of the transcription cycle [92, 93] is evident from the observation that depletion of INTS11 altered Pol II chromatin-binding kinetics even under unperturbed conditions (Fig. 4C). INTS11 depletion resulted in an increased Pol II immobilization, suggesting that the pool of transcriptional engaged, chromatin-bound Pol II is increased upon INTS11 depletion. These findings might be explained by the fact that loss of Integrator reduces both promoter-proximal pausing and premature transcription termination, resulting in more chromatin-bound Pol II [63]. For this study, we especially focused on the effects of INTS11 depletion on Pol II chromatin binding upon DNA damage. In line with previous results [25, 48], UV-induced DNA damage increased Pol II immobilization, especially of the long-bound elongating fraction, as evident from the reduced slope of the FRAP curve at timepoints >100 s (Fig. 4C). This increased residence time of elongating Pol II likely reflects slower elongating Pol II due to stalling at TBLs. Strikingly, upon INTS11-depletion, the immobile Pol II fraction increased by ∼50% following UV exposure compared to WT cells, indicating an accumulation of lesion-stalled Pol II in the absence of INTS11. Of note, total Pol II levels as determined by GFP-RPB1 fluorescence were similarly reduced in the presence or absence of UV irradiation (Supplementary Fig. S4B), consistent with flow cytometry data (Fig. 4A and Supplementary Fig. S4A). Together, these findings indicate that INTS11 promotes the removal of chromatin-bound Pol II during TC-NER, as its depletion stabilizes the pSer2-modified transcriptionally engaged Pol II fraction following UV irradiation.

### INTS11 promotes removal of Pol II at TC-NER damage sites

The increased chromatin-binding of transcriptionally engaged Pol II in the absence of the Integrator complex upon DNA damage could be explained by prolonged stalling of Pol II, but may also result from elongating Pol II molecules that accumulate or stall upstream from lesion-stalled Pol II complexes [22, 50]. To more directly assess the residence time of lesion-stalled Pol II, we used an imaging-based Pol II DRB run-off assay to monitor the chromatin release of lesion-stalled Pol II [29, 73, 94] (Fig. 5A). This assay enables visualization of retention or eviction of pre-existing elongating Pol II complexes on the chromatin following local UV damage. Local DNA damage was induced in cell nuclei by UV irradiation through a microporous filter [95]. To selectively monitor the fate of pre-existing lesion-stalled Pol II complexes, 5,6,dicloro-1-β-D-ribofuranosylbenzimidazole (DRB) was applied immediately after UV irradiation to block interference from *de novo* transcription initiation [96]. Clearance of lesion-stalled Pol II was then evaluated over time by IF staining of pSer2-Pol II. In control cells, pSer2-Pol II levels at damage sites, visualized by CPD staining, declined over time, due to TC-NER-mediated TBL repair (Fig. 5B). In contrast, in TC-NER deficient CSB-depleted cells, lesion-stalled Pol II remained prolonged chromatin-bound, consistent with defective clearance of lesion-stalled Pol II by TC-NER or by alternative CRL4^CSA^-mediated Pol II degradation [22, 49, 50, 73] (Fig. 5B and Supplementary Fig. S5A). Interestingly, in siRNA-mediated INTS11-depleted cells, removal of lesion-stalled Pol II was delayed, yielding an intermediate phenotype. Similar results were obtained using PROTAC3-mediated INTS11 depletion (Supplementary Fig. S5E), showing that the Integrator complex is important for the release of initially lesion-stalled Pol II.

**Figure 5.**
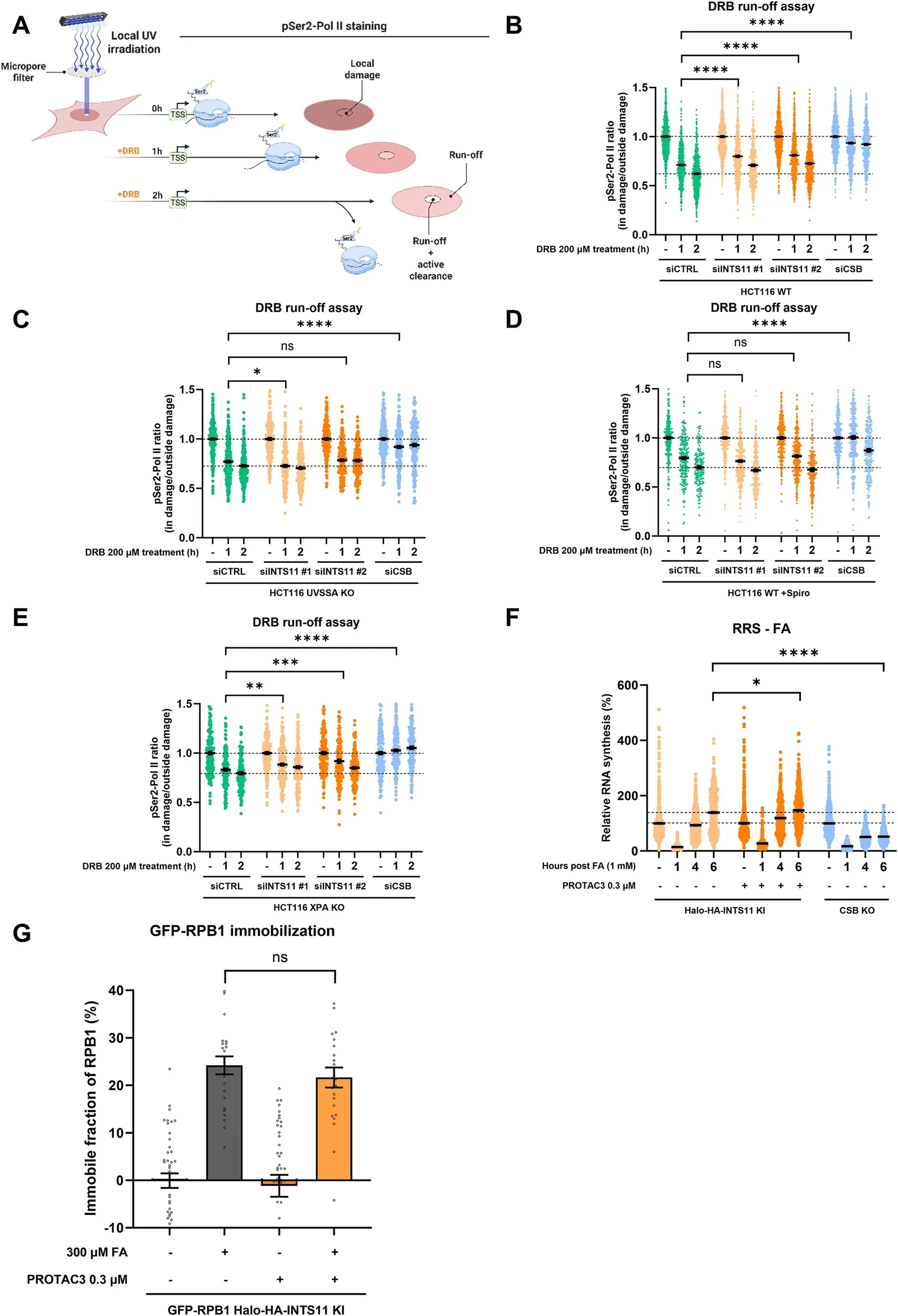
Integrator complex stimulates chromatin release of lesion-stalled Pol II. (**A**) Schematic cartoon of the Pol II DRB run-off assay to compare the release of lesion-stalled Pol II at sites of local damage, with that of non-damage stalled Pol II outside of the local damage. Cells were irradiated with UV through a micropore filter to induce local DNA damage. New transcription initiation was blocked with the CDK9 inhibitor DRB, and the removal of elongating Pol II at sites of DNA damage, as determined by a CPD staining, was compared to the removal outside UV-damaged areas using pSer2-modified Pol II IF. (**B**) pSer2-modified elongating Pol II signal quantification at sites of local UV-induced DNA damage in HCT116 cells upon depletion of the indicated proteins using siRNAs. Cells were locally irradiated with UV (80 J/m^2^) and treated with DRB immediately following UV irradiation and IF was performed at the indicated times after UV. CPD staining was used to identify areas of local UV damage. The mean pSer2-modified Pol II intensity within the damaged region was divided by the mean intensity outside the damaged region in the same nucleus to normalize for the overall reduction in pSer2-modified Pol II signal caused by DRB-induced transcription inhibition resulting in a Pol II run-off. A ratio <1 indicates that pSer2-modified Pol II is released faster at sites of local damage than the general transcription run-off in undamaged regions. Black lines indicate average pSer2-Pol II ratio ± SEM from three independent experiments (*n* ≥ 437 cells per condition). Statistical significance was determined using an unpaired two-tailed Student’s *t*-test (**** *P < *.0001). Pol II-Ser2 signal quantification at sites of local UV-induced DNA damaged as in panel (**B**) in HCT116 (**C**) UVSSA KO, (**D**) spironolactone-treated WT (10 µM, 2 h), and (**E**) CSB-mScarletI KI XPA KO cells upon depletion of the indicated proteins using siRNAs. Black lines indicate average pSer2-Pol II ratio ± SEM from three independent experiments (panel (**C**) *n* ≥ 197 cells, panel (**D**) ≥ 164, and panel (**E**) *n* ≥ 155 cells per condition). Statistical significance was determined using an unpaired two-tailed Student’s *t*-test (ns = non-significant, *P < .05, **P < .01, ***P < .001, **** *P < *.0001). (**F**) Quantification of nascent transcription levels as determined by EU incorporation (30 min) at the indicated timepoints after FA treatment (1 µM, 30 min) in HCT116 Halo-HA-INTS11 KI and HCT116 CSB KO. Where specified, cells were pre-treated with PROTAC3 (0.3 µM, 16 h) to deplete INTS11. Mean RFI of EU (relative RNA synthesis) were normalized to untreated levels and set to 100% (dotted line). Black lines indicate average integrated density ± SEM from three independent experiments (*n* ≥ 596 cells per condition). Statistical significance was determined using an unpaired two-tailed Student’s *t*-test (*P < .05, ****P < .0001). (**G**) Calculated immobile fraction of GFP-RPB1 in mock- and FA-treated (300 µM, 30 min) RPE1 GFP-RPB1 Halo-HA-INTS11 KI cells from the data points in the dotted box indicated in Supplementary Fig. S5H (timepoints 219.4 s–259.4 s). PROTAC3 (0.3 µM, 24 h) pre-treatment was done when indicated. Immobile fraction in WT and INTS11-depleted cells was calculated by comparing RFI after FA to mock-treated sample and normalizing to their own non-treated RFI values. *n* ≥ 24 cells per condition from at least three independent experiments ± SEM. Statistical significance was determined using an unpaired two-tailed Student’s *t*-test (ns, non-significant). Panel (A) (https://biorender.com/cqe816m) was created using BioRender.

Many TBLs, such as UV-induced CPDs and 6–4PPs, are fully shielded by the stalled Pol II complex, preventing direct access of the TC-NER incision machinery [37, 38]. Several, non-mutually exclusive mechanisms have been proposed to expose these lesions. One model suggests Pol II backtracking, most likely driven by the TFIIH subunit XPD through its 5′–3′ translocase activity, allowing lesion access while Pol II remains DNA-bound [3, 31, 39, 41, 42]. Alternatively, Pol II may be released via premature transcription termination [43, 45] or targeted for proteasomal degradation [19, 23, 49, 50, 73]. Our finding that INTS11 depletion does not impair TC-NER (Fig. 3), but delays the release of the initially lesion-stalled Pol II from chromatin (Fig. 5B), indicates that Integrator stimulates Pol II release after lesion repair, rather than during lesion exposure. These findings support a two-step Pol II processing mechanism in which first Pol II needs to be processed to expose the lesion to allow repair. Because TC-NER proceeds normally in the absence of INTS11, while the initially lesion-stalled Pol II remains chromatin-bound, lesion exposure must occur without Pol II dissociation, making TFIIH-mediated Pol II backtracking the most plausible mechanism to expose the lesion for repair. Subsequently, in a second step following repair, the Integrator complex promotes release of this possible backtracked Pol II from the chromatin to facilitate efficient transcription recovery.

This two-step Pol II processing model suggests that when Pol II backtracking cannot occur, INTS11 will not affect Pol II release from the chromatin. To test this model, we first examined the role of the Integrator complex on Pol II release in UVSSA KO cells, in which STK19 and TFIIH recruitment to the TC-NER complex is impaired [17, 27, 28, 31] and therefore also recruitment of the Integrator complex. In this situation, Pol II release should be independent of the Integrator complex. As expected, INTS11 depletion in UVSSA KO cells did not impede Pol II release (Fig. 5C and Supplementary Fig. S5B). This further suggests that in TC-NER-deficient UVSSA KO cells, lesion-stalled Pol II is cleared independently of Integrator, likely through CRL4^CSA^-mediated proteasomal degradation as an alternative mechanism for Pol II removal [2, 3, 22, 39, 49, 50, 73]. Consistent with this, INTS11 depletion did not increase chromatin-bound pSer2 Pol II levels in UVSSA KO cells (Supplementary Fig. S5F), as was observed in WT cells (Fig. 4B).

To further test our model, we examined the role of Integrator in Pol II release following acute TFIIH depletion by treating cells with spironolactone, which induces rapid degradation of the TFIIH subunit XPB [76]. Consistent with our model, INTS11 depletion did not further impair Pol II release in spironolactone-treated cells (Fig. 5D and Supplementary Fig. S5C), supporting the notion that Integrator functions downstream of TFIIH during Pol II processing. In contrast to spironolactone-treated cells, TFIIH recruitment remains intact in XPA KO cells [17]. Biochemical studies have shown that, although XPA is essential for damage verification and excision, TFIIH retains residual translocase activity in its absence *in vitro* [97, 98]. Accordingly, it has been shown that Pol II is efficiently removed from damaged chromatin in a degradation-independent manner, presumably via a similar mechanism as in TC-NER proficient cells [50, 73]. Interestingly, INTS11 depletion in XPA KO cells leads to a prolonged retention of lesion-stalled Pol II, to a similar extent as observed in WT cells (Fig. 5E and Supplementary Fig. S5D). This suggests that Pol II release by the Integrator complex requires TFIIH-mediated Pol II backtracking. Alternatively, Integrator may recognize a stalled, non-productive TFIIH-containing repair intermediate rather than specifically a backtracked Pol II complex. Interestingly, despite the TC-NER deficiency in XPA KO cells, Pol II can still be released from the chromatin by the Integrator complex, indicating that lesion removal is not a prerequisite for INTS11-dependent Pol II release.

To further examine our model that Integrator acts downstream of TFIIH-mediated Pol II backtracking, we examined the role of the Integrator complex during transcription-coupled repair of DNA–protein crosslinks (TC-DPCR). DPCs are structurally diverse lesions formed by covalent linkage of proteins to DNA via enzymatic or non-enzymatic processes, such as those induced by formaldehyde (FA) [99]. Unlike bulky UV-induced lesions [37, 38], DPCs are large adducts that obstruct Pol II before the lesion reaches its active site [10–12, 100]. Transcription-blocking DPCs are resolved by a dedicated TC-DPC repair pathway, which requires the action of CSB and CRL4^CSA^, but acts independently of UVSSA and TFIIH [10–12, 100]. Consequently, backtracking of Pol II is not expected to occur during TC-DPCR. Because our data indicate that INTS11-mediated Pol II release depends on the TFIIH pathway and most plausibly acts downstream of Pol II backtracking, we hypothesized that the Integrator complex would not contribute to Pol II removal during TC-DPCR. To test this, we determined the recovery of transcription after FA-induced DPCs. After FA-induced transcription inhibition, transcription swiftly recovered in WT cells with a full recovery within 4 h (Fig. 5F). As expected, transcription recovery was abolished in TC-DPCR-deficient CSB KO cells. Notably, in contrast to UV or Illudin S-induced damage (Fig. 2C–F), INTS11 depletion had no effect on transcription resumption following FA-induced damage. Similarly, FRAP analysis showed that FA-induced Pol II immobilization was comparable in WT and INTS11-depleted cells (Fig. 5G and Supplementary Fig. S5G and H), consistent with previous observations in WT cells [12]. In contrast, under identical experimental conditions, INTS11 depletion increased UV-induced Pol II immobilization by ∼50% (Fig. 4C). Together, these findings support our model that Integrator functions specifically downstream of the TFIIH-dependent processing of initially lesion-stalled Pol II and is therefore likely dispensable during TC-DPCR.

Together, our data extend the known roles of Integrator beyond its well-established functions in regulating multiple stages of the transcription cycle under unperturbed conditions, revealing an essential role during DNA damage-induced transcription stress in ensuring timely restoration of gene expression. We identified the Integrator complex as a critical effector of post-repair transcription recovery, functioning downstream of TC-NER to actively release previously lesion-stalled Pol II from chromatin to facilitate efficient transcription recovery.

## Discussion

Although our understanding of the TC-NER mechanism has greatly advanced in recent years [39, 101, 102], how exactly transcription recovers following repair has remained unclear. This is an important question, as even when transcription-blocking DNA damage is efficiently removed by TC-NER, its initial presence remains likely cytotoxic if the essential transcription process is not properly recovered. It was initially assumed that transcription would resume directly from the repair site [8, 103], a scenario that requires Pol II to stay bound to the DNA template during repair. However, recent specialized sequencing and modeling studies suggested that transcription restart from the promoter, by *de novo* transcription initiation, is likely the predominant restart mechanism [43, 44]. Correspondingly, genome-wide nascent RNA-seq analyses have shown that transcription recovery often proceeds as a wave originating from the promoter [104, 105]. This promoter-driven restart is consistent with the findings that transcription restart can be facilitated by the PAF1 complex, which promotes processive transcription following DNA damage, by stimulating Pol II pause–release at promoter-proximal regions [105]. These findings suggest that lesion-stalled Pol II is released from the DNA during the cellular responses to DNA damage-induced transcription stress. Indeed, our data show that in WT cells, Pol II is released from chromatin more rapidly at sites of DNA damage than under undamaged conditions (Fig. 5A and B), consistent with recently published studies [50, 73]. Together, this suggests that the removal of lesion-stalled Pol II from the chromatin is the most prevalent scenario. Nonetheless, the possibility that transcription can also resume locally at the repair site cannot be ruled out.

Here, we show that the Integrator complex, although recruited to the TC-NER complex (Fig. 1), is not essential for TC-NER-mediated repair (Fig. 3). Instead, INTS11 functions downstream of the repair reaction, where it is required for transcription recovery following TBL repair (Fig. 2) by facilitating clearance of initially lesion-stalled Pol II from the chromatin (Figs 4 and 5). These findings revealed a thus far unknown role for the Integrator complex in the processing of lesion-stalled Pol II to allow efficient transcription restart.

Processing of lesion-stalled Pol II is critical for the proper execution of TC-NER [2, 3, 39]. Before TFIIH can perform its damage verification function and mediate lesion excision, the lesion-stalled Pol II must be processed to expose the TBL. This is because most TBLs, including UV-induced CPDs and 6–4PPs [37, 38], reside within the Pol II catalytic core and are therefore inaccessible to the downstream repair factors of the incision complex [37, 106]. In addition, the presence of lesion-stalled Pol II likely sterically obstructs DNA incision by the endonuclease XPG [107]. Currently, three non-mutually exclusive models have been proposed that can enable Pol II clearance to expose the lesion. One proposed mechanism suggests that Pol II is cleared through Pol II ubiquitylation, subsequent chromatin extraction by the ubiquitin-selective segregase VCP/p97, followed by proteasomal degradation [23, 46–48]. However, recently it was shown that Pol II degradation is not the primary mode of Pol II processing during TC-NER and therefore, acts likely as a last-resort pathway if TC-NER fails [49–51, 73]. Another model poses that Pol II is backtracked (reverse translocated), exposing the TBL while Pol II remains bound to the DNA template [2, 39, 41, 42]. TFIIH is a likely driver for such Pol II backtracking, as predicted structural cryo-EM models place the XPD helicase of TFIIH in an ideal position to employ its 5′-3′ translocase activity [27, 29, 30]. In this configuration, XPD could exert a pulling force that draws the lesion away from the Pol II active site toward TFIIH to allow damage verification and subsequent TBL excision, thereby promoting reverse movement of Pol II. Alternatively, other models suggest that Pol II is released from the DNA, for instance by premature transcription termination [43–45].

Our data suggest that the Integrator complex facilitates the resolution of initially lesion-stalled Pol II (Figs 4 and 5), indicating that this represents the predominant mechanism for clearing stalled transcription complexes. This Pol II clearance depends on the endonuclease activity of the Integrator complex (Figs 1 and 2D and E). The Integrator nuclease activity mediates RNA cleavage during snRNA processing [53] and promotes termination of stalled or excessively paused Pol II through cleavage of the nascent RNA and facilitates its degradation, which likely destabilizes the DNA–RNA hybrid and collapses the transcription bubble, thereby releasing Pol II from the DNA [60, 64, 66, 108, 109]. We propose that a similar mechanism operates during TC-NER, where the Integrator endonuclease cleaves the nascent RNA to promote the release of previously lesion-stalled Pol II. If unresolved, such persistent chromatin-bound Pol II complexes would be expected to create a physical barrier for newly initiated RNA polymerases during promoter-driven transcription restart, thereby delaying recovery despite successful lesion repair. This model is supported by our findings that INTS11 depletion causes a strong transcription recovery defect despite only a modest impairment in initial Pol II clearance (Figs 2 and 5). While DRB run-off assays reveal a limited delay in clearance of the initially lesion-stalled Pol II (Fig. 5), phospho-Ser2 Pol II chromatin-binding and FRAP analyses demonstrate a much more pronounced accumulation of chromatin-bound transcriptional engaged Pol II (Fig. 4), indicating that persistent Pol II complexes, due to incorrect chromatin release, may result in Pol II traffic jams and constitute a major barrier to efficient transcription recovery.

Interestingly, this defect in transcription recovery occurs independently of a TC-NER repair defect, since it does not affect TC-NER-mediated TBL removal, as TC-NER complex assembly, lesion incision, and repair synthesis remain fully intact in its absence (Fig. 3). This is an important observation, as it suggests that Integrator-mediated Pol II release is not the primary Pol II processing mechanism to expose the TBL for repair, but instead functions after TC-NER to remove Pol II from the DNA and ensure proper transcription recovery (Fig. 6). Interestingly, a recent study identified INTS12, a non-catalytic component of the backbone module of the Integrator complex that has been implicated in ribotoxic stress signaling, as an important regulator of transcription recovery following UV-induced DNA damage [110]. Consistent with our findings, loss of INTS12 delayed Pol II clearance and impaired transcription recovery following UV-induced DNA damage, whereas no effect was observed during TC-DPCR. In contrast to our findings, however, loss of INTS12 also impaired TC-NER despite unaffected TC-NER complex assembly and Pol II ubiquitylation. These findings show that individual modules of the Integrator complex fulfill distinct functions during the TC-NER response and further underscore the multifunctional role of the Integrator complex not only during transcription regulation but also during TC-NER.

**Figure 6.**
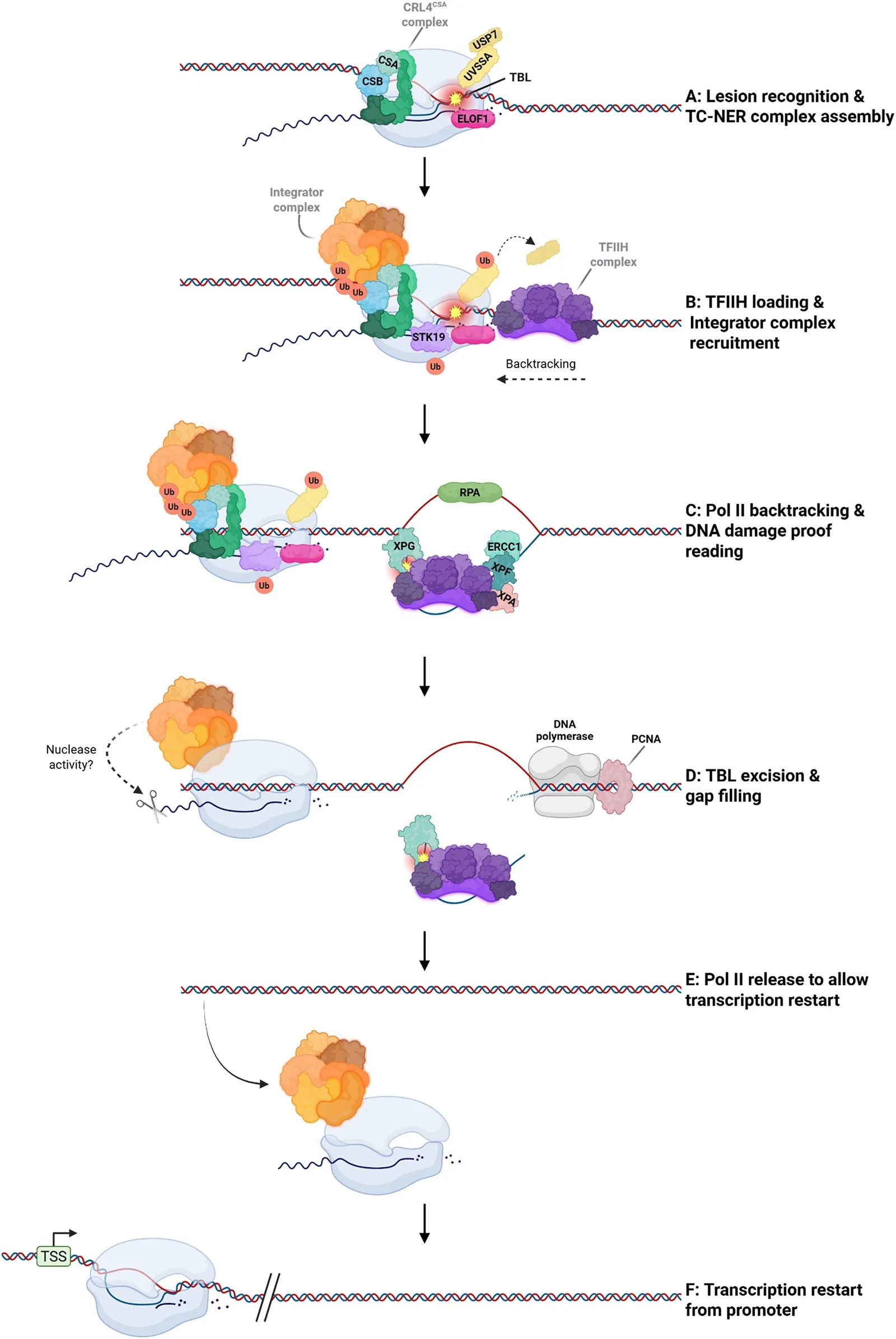
Integrator complex stimulates a two-step mechanism of Pol II processing at TBLs. Model showing the Integrator complex function during the cellular response to transcription-blocking DNA damage. (**A**) Transcription-blocking lesions block Pol II-meditated transcription, by blocking the Pol II forward movement in the active site of Pol II. As a consequence Pol II shields the damage. After detection of lesion-stalled Pol II the TC-NER complex is assembled. (**B**) The Integrator complex is recruited during a late stage of the TC-NER reaction, downstream of STK19 recruitment at the reaction step in which TFIIH is recruited and correctly positioned for damage proofreading. (**C**) For correct handover of the lesion from the TC-NER initiating complex to the TC-NER incision complex, Pol II needs to be removed from the lesion, while remaining bound to the DNA. The likely scenario is that Pol II is backtracked by the 5′-3′ translocase activity of the TFIIH subunit XPD. (**D**) Pol II backtracking allows TBL removal by the incision complex and subsequent gap filling of the ssDNA gap. (**E**) In parallel, the catalytic core of the Integrator complex stimulates chromatin release of the backtracked Pol II, which would otherwise formed persistent roadblocks for newly initiated elongation complexes. (**F**) By removing the initially lesion-stalled Pol II, INTS11 facilitates proper transcription recovery by *de novo* transcription initiation from the transcription start site (TSS). Fig. 6 (https://biorender.com/pae7et5) was created using BioRender.

In line with our model that Integrator functions after TC-NER to remove Pol II from the DNA to ensure proper transcription recovery, in TFIIH-depleted cells, no effect of the Integrator complex on Pol II release could be observed (Fig. 5D). This Pol II release downstream of TC-NER-mediated excision suggests that the Integrator complex does not remove the actual lesion-stalled Pol II, but rather removes an already processed (most plausible backtracked) Pol II in a secondary response to allow transcription recovery. Importantly, this Pol II removal model furthermore indicates that during the 1st Pol II processing step, Pol II remains DNA bound. This is consistent with a model that the first Pol II processing step involves backtracking, as this is thus far the only known mechanism that can expose the lesion while keeping Pol II engaged to the DNA. Thus far, Pol II backtracking to expose the lesion during TC-NER was assumed to be unlikely. First of all, because restart from the lesion site was not commonly observed [43, 44, 104, 105]. Secondly, because re-activation of such extensively backtracked Pol II complexes would require TFIIS, which is not recruited to the TC-NER complex and is dispensable for TC-NER [11]. The absence of TFIIS involvement during TC-NER can be explained by the fact that UVSSA contains a TFIIS-like element that competes with TFIIS for interaction with lesion-stalled Pol II, preventing TFIIS-mediated reactivation of backtracked Pol II complexes [111].

Our study supports a two-step model of Pol II processing in which TFIIH-mediated Pol II backtracking first exposes the lesion for repair, after which the Integrator complex promotes release of the previously lesion-stalled Pol II from chromatin, most likely through premature transcription termination. This enables transcription to restart from the promoter without the initially lesion-stalled Pol II acting as a persistent barrier to newly initiated RNA polymerases (Fig. 6). This two-step mechanism also explains why backtracked Pol II can overcome without the need for TFIIS, as the backtracked Pol II is not reactivated, but rather released from the DNA in the second step (Fig. 6). Moreover, this model is in line with previous sequencing and modeling studies showing that transcription re-initiates primarily at the promoter following UV-induced transcriptional arrest due to Pol II chromatin release [43, 44].

Of note, depletion of the Integrator complex has only a partial effect on Pol II removal. This suggests that additional Pol II clearance mechanisms may act in parallel, for instance other transcription termination factors such as XRN2, TTF2, PCF11, and RPRD1B, the latter three of which have been implicated in the transcription stress response to UV-induced DNA damage [45, 52]. These backtracked Pol II complexes, together with efficient TBL removal by TC-NER, may also adopt a less stable or more transiently bound state than lesion-stalled Pol II, which could explain the more severe Pol II stalling observed in TC-NER-deficient CSB KO cells (Fig. 5B). Alternatively, Pol II might be targeted for p97/VCP-mediated degradation [73], although no clear increase in Pol II ubiquitylation or degradation was observed upon INTS11 depletion, suggesting that this is not likely to occur.

In line with its proposed function downstream of repair, our data show that the Integrator complex is recruited at a late stage of the TC-NER, downstream of STK19 and TFIIH recruitment (Fig. 1). This indicates that the Integrator complex is recruited to lesion-stalled Pol II at the same stage, or possibly even after correct TFIIH incorporation into the TC-NER complex. This reaction step, preceding TFIIH-mediated Pol II backtracking [2, 112], likely represents the final stage at which TFIIH remains associated with lesion-stalled Pol II. Consequently, this defines the last opportunity for the Integrator complex to be specifically loaded onto lesion-stalled Pol II complexes, distinguishing it from other forms of persistently or long-term paused Pol II complexes. Moreover, recruitment of the Integrator complex at later stages of the TC-NER reaction, e.g. to the incision complex, would not allow for the discrimination of TFIIH engaged in either GG-NER or TC-NER. This STK19- and TFIIH-dependent recruitment of the Integrator complex likely also explains why in TC-NER-deficient XPA KO cells, in which TFIIH is still properly recruited and positioned and was described to retain 5′-3′ residual translocase activity *in vitro*, Integrator complex-dependent release of backtracked Pol II complexes might still occur (Fig. 5E). Interestingly, this observation indicates that although TC-NER can proceed in the absence of Integrator complex-mediated Pol II release, repair by TC-NER itself is not a pre-requisite for INTS11-mediated Pol II dissociation, but rather the Pol II backtracking is the trigger. Although our data support a model in which Integrator acts on backtracked Pol II to promote its clearance, based on our data we cannot exclude an alternative mechanism in which Integrator recognizes a stalled, non-productive TC-NER intermediate containing correct assembled TFIIH and downstream repair factors, independent of productive Pol II backtracking. This scenario is likely only relevant under specific conditions like in the absence of TC-NER, such as in XPA KO cells, where stabilized repair intermediates could provide an alternative platform for Integrator recruitment.

Notably, this role of the Integrator complex in transcription recovery is specific for DNA damage, as it was not involved in transcription recovery upon removal of the reversible CDK9 inhibitor flavopiridol (Fig. 2G). Moreover, the Integrator complex is also specific for the type of transcription-blocking lesion. While the Integrator complex is important for the transcription restart upon UV and Illudin S-induced bulky lesions (Fig. 2C–F), it is not involved upon restart after FA-induced transcription-blocking DPCs (Fig. 5F). Also, in contrast to UV-induced damage, Pol II stalling at DPCs is unaffected by loss of INTS11 (Fig. 5G). This dependency for specific lesion-types that block Pol II is also in line with our two-step Pol II processing model. In contrast to UV-induced damage, DPCs will block Pol II without entering the active site of Pol II [113]. As a consequence, no Pol II backtracking is needed to expose and repair the DPC lesion by proteasomal degradation mediated by CRL4^CSA^-mediated ubiquitylation of the DPC [10–12]. In line with the absence of Pol II backtracking and lesion excision during TC-DPCR, TFIIH, XPA and the endonucleases XPG and XPF/ERCC1 are not needed. As during TC-DPCR no extensive Pol II backtracking occurs, there is also no need to remove the backtracked Pol II by the Integrator complex to allow transcription recovery. In contrast, during TC-DPCR, TFIIS is recruited to the DPC-stalled Pol II complex to facilitate transcription resumption, likely form the damage site [11].

Thus, two damage-specific modes of Pol II processing exist depending on the lesion type: during TC-NER, TBLs stall Pol II within its active site, necessitating Pol II backtracking followed by Integrator-mediated release to facilitate proper transcription recovery of *de novo* transcription events from the promoter. In contrast, for transcription-blocking DPCs, which likely obstruct Pol II before it reaches the active site, backtracking is unnecessary, and TFIIS allows Pol II to resume elongation directly from the repair site. These lesion-specific processing modes likely explain why transcription recovery occurs more rapidly during TC-DPCR (∼6 h) than during TC-NER (≥16 h), reflecting the difference between resuming elongation at the lesion site versus reinitiating from the promoter.

Together, our findings extend the known roles of the Integrator complex beyond its functions during normal transcription, revealing a critical role during DNA damage-induced transcription stress. INTS11 acts downstream of TC-NER to release initially lesion-stalled Pol II from chromatin. If not removed, such persistent arrested Pol II complexes might act as a roadblock for newly initiated elongation complexes, thereby impeding efficient promoter-driven transcription restart. While dispensable for the repair of TBLs themselves, Integrator’s endonuclease activity is essential for resolving previously lesion-stalled Pol II. The proposed two-step Pol II processing model, consisting of TFIIH-mediated Pol II backtracking followed by Integrator-dependent chromatin release, adds another regulatory layer to the intricate cellular responses to transcription-blocking DNA damage and positions Integrator as a central regulator of transcriptional homeostasis both in unperturbed conditions and after transcription-blocking DNA damage.

## Supplementary Material

gkag1068_Supplemental_File

## Data Availability

Proteomics data are deposited at the ProteomeXchange Consortium through the PRIDE partner repository with dataset identifier PXD051720.
