# Supplementary material for: Integrator complex removes lesion-stalled RNA polymerase II after transcription-coupled repair to facilitate transcription restart
Source: Nucleic Acids Res. 2026 Oct 31;54(20):gkag1068. doi: 10.1093/nar/gkag1068 (PMC13722877; doi:10.1093/nar/gkag1068)
Supplement: Supplementary file 1 [file gkag1068_supplemental_file.pdf]

# Supplemental Figure 1 Belonging to figure 1

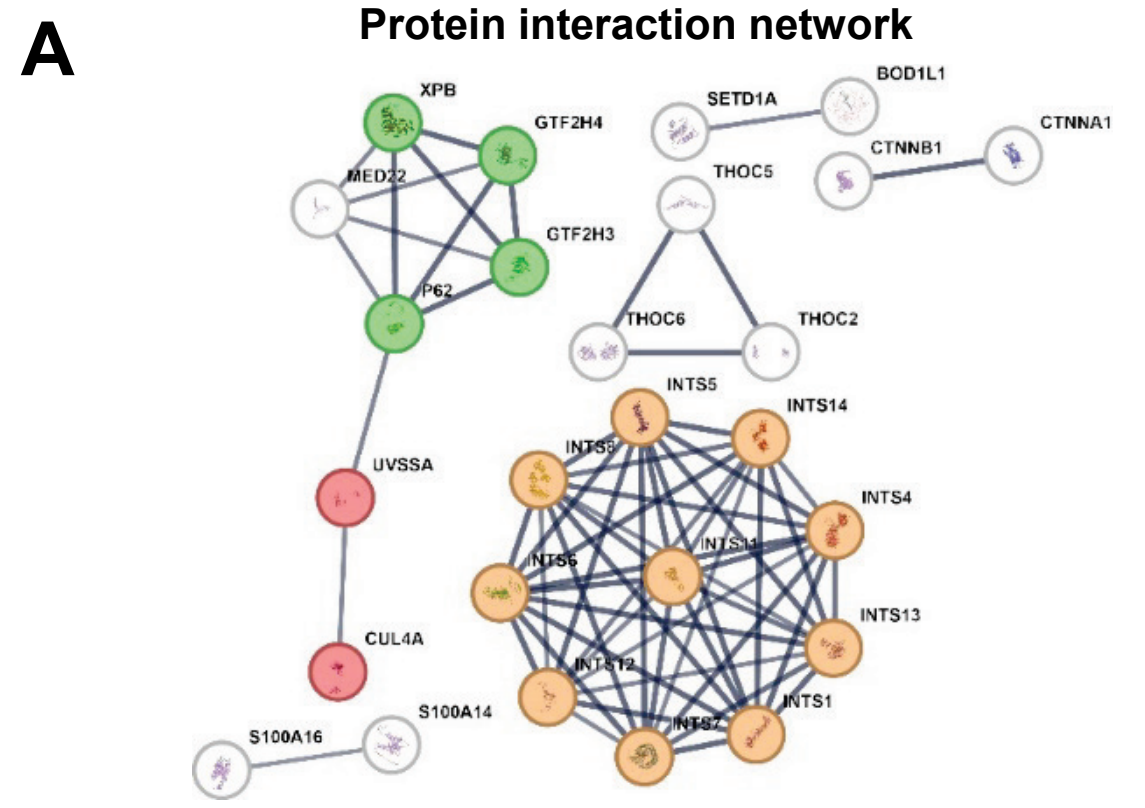


B

| GO term: Biological processes | GO term ID | Signal | False discovery rate |
|---|---|---|---|
| snRNA processing | GO:0016180 | 3.9 | 1.96E-13 |
| Regulation of DNA-templated transcription, elongation | GO:0032784 | 3.89 | 9.12E-16 |
| snRNA metabolic process | GO:0016073 | 3.62 | 2.11E-13 |
| Regulation of transcription elongation from RNA Polymerase II promoter | GO:0034243 | 3.45 | 1.96E-13 |
| snRNA 3-end processing | GO:0034472 | 2.52 | 1.03E-08 |

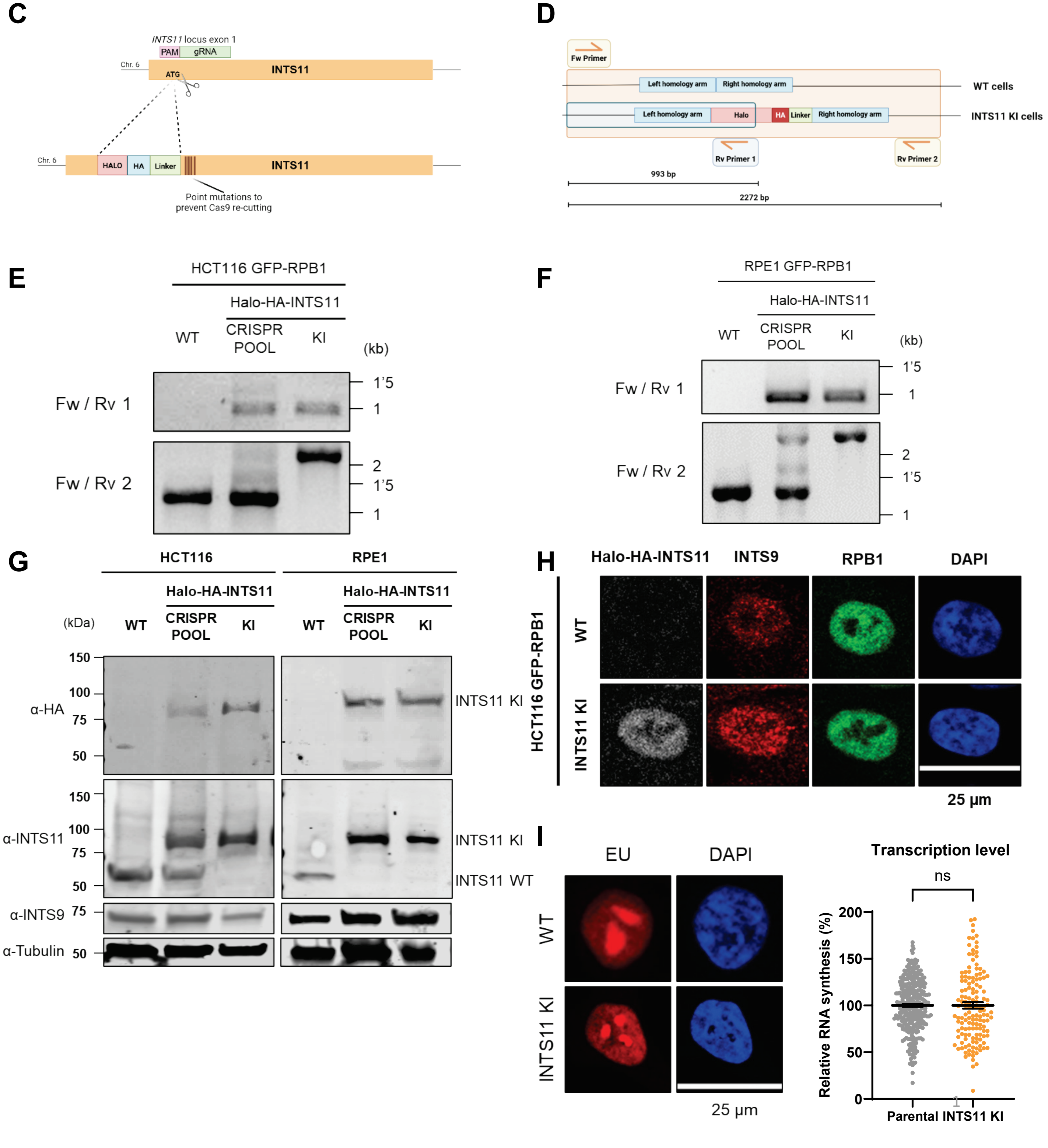

**Supplemental Figure 1 Belonging to figure 1**

**A:** STRING protein-protein interaction network of the STK19-dependent pSer2-Pol II interactors upon UV-induced damage as shown in Fig. 1A, with a Log2 SILAC ratio > 0.6.

**B:** Top 5 enriched GO terms (biological process) identified using g:Profiler of the STK19-dependent pSer2-Pol II interactors upon UV-induced damage as shown in Fig. 1A, with a Log2 SILAC ratio > 0.6.

**C:** Schematic cartoon depicting insertion of a Halo-HA Tag at the N-terminal of endogenous INTS11.

**D:** Schematic representation of PCR-based genotyping for Halo-HA knock-in (KI). Primer pair 1 (Fw-Rv1) was used to identify potential KI clones, while primer pair 2 (Fw-Rv2) allowed discrimination between homozygous and heterozygous KI alleles.

**E, F:** PCR of genomic DNA indicating homozygous integration of the Halo-HA tag in the CRISPR pool and of the positive clone in HCT116 (**E**) or RPE1 (**F**) in GFP-RPB1 Halo-HA-INTS11 KI cells. A parental cell line is used as negative control.

**G:** Immunoblot analysis using the indicated antibodies in WT HCT116 GFP-RPB1 KI cells, or Halo-HA-INTS11 KI cells and RPE1 WT cells, or Halo-HA-INST11 KI cells. Tubulin was used as a loading control.

**H:** Representative immunofluorescence (IF) images showing colocalization of endogenously expressed Halo-HA-INTS11 and Pol II (GFP-RPB1) in HCT116 GFP-RPB1 KI WT cells and Halo-HA-INTS11 KI cells. Scale bar, 25 µm.

**I:** (Left) IF analysis and (Right) quantification of nascent transcription levels as determined by EU incorporation in HCT116 WT and Halo-HA-INTS11 KI cells, showing comparable transcription levels. Black bar represents average EU levels. Scale bar, 25 µm.

Panels (C) (https://BioRender.com/clbiyk9) and (D) (https://BioRender.com/d0cc338) were created in BioRender.

# Supplemental Figure 2 Belonging to figure 2

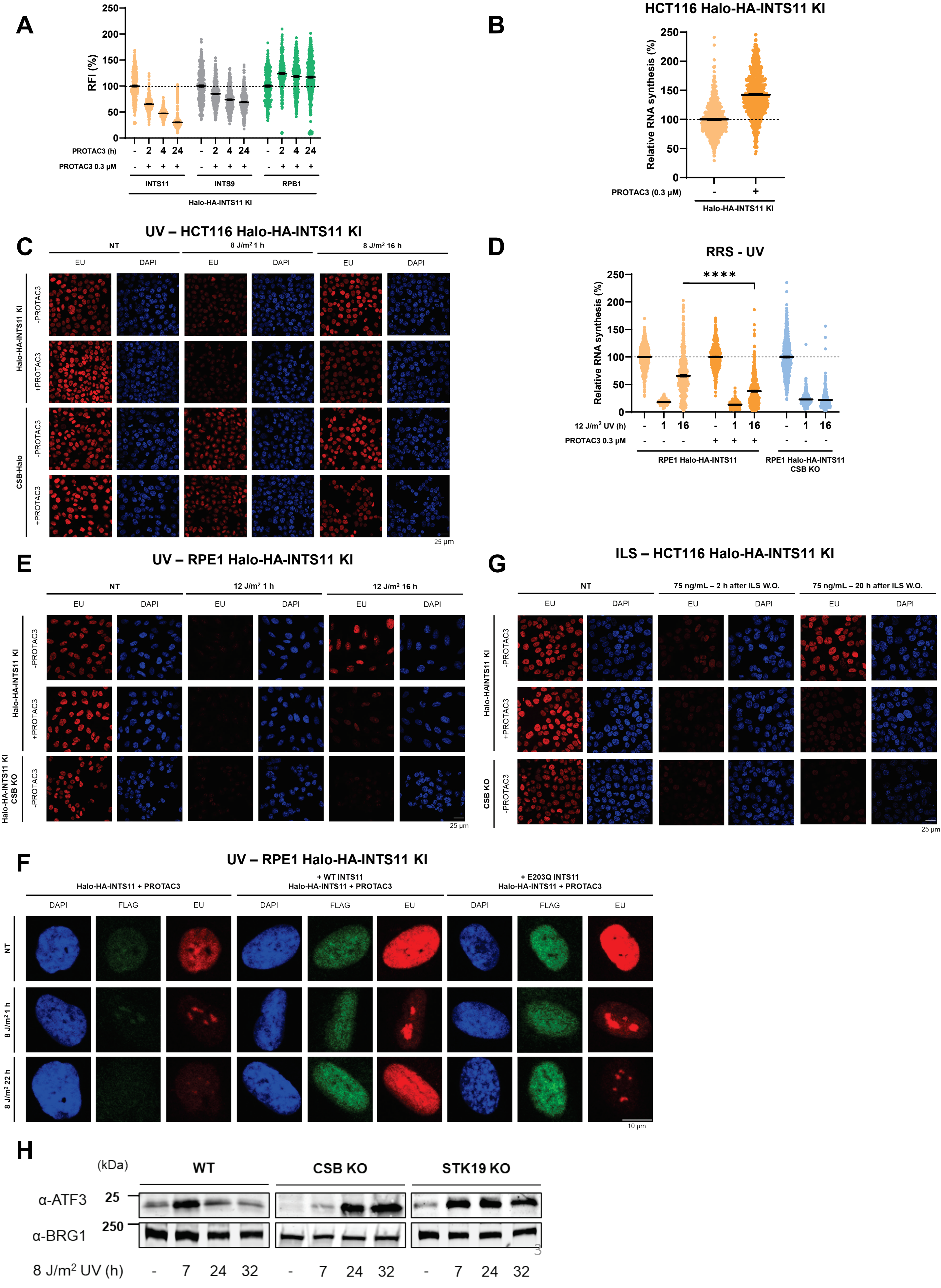

**Supplemental Figure 2 Belonging to figure 2**

**A:** Quantification of INTS11, INTS9 and RPB1 protein levels following treatment with PROTAC3 (0.3 µM) for the indicated time points, as assessed by immunofluorescence in HCT116 GFP-RPB1 Halo-HA-INTS11 KI cells.

**B:** Quantification of nascent transcription levels after INTS11 depletion based on relative EU incorporation in Halo-HA-INTS11 KI cells in mock- and PROTAC3- (0.3 µM, 16 h) treated cells. EU fluorescence intensity was normalized to mock-treated levels (set to 100%). Black lines indicate average integrated density ± SEM from three independent experiments (n ≥ 616 cells per condition).

**C:** Representative images of nascent transcription levels as determined by relative EU incorporation in irradiated and non-irradiated HCT116 Halo-HA-INTS11 KI cells and HCT116 CSB-Halo 1 h and 16 h after UV exposure (8 J/m$^2$). Where indicated cells were treated with PROTAC3 (0.3 µM, 16 h) to deplete INTS11 or CSB. Quantification is shown in Fig. 2C. NT, not treated.

**D:** Quantification of nascent transcription levels as determined by EU incorporation (30 min) at the indicated timepoints after UV irradiation (8 J/m$^2$) in RPE1 Halo-HA-INTS11 KI cells and RPE1 Halo-HA-INTS11 KI CSB KO cells. Where specified, cells were pre-treated with PROTAC3 (0.3 µM, 16 h) to deplete INTS11. Mean relative fluorescence intensities of EU (relative RNA synthesis) were normalized to untreated levels and set to 100% (dotted line). Black lines indicate average integrated density ± SEM from three independent experiments (n ≥ 430 cells per condition). Statistical significance was determined using an unpaired two-tailed Student's t-test (****P < 0.0001).

**E:** Representative images of transcription levels as determined by relative EU incorporation in irradiated and non-irradiated RPE1 Halo-HA-INTS11 KI cells and RPE1 Halo-HA-INTS11 KI CSB KO 1 h and 16 h after UV exposure (12 J/m$^2$). Where indicated cells were treated with PROTAC3 (0.3 µM, 16 h). NT, not treated. Quantification is shown in Supplementary Fig. 2D.

**F:** Representative images of transcription levels as determined by relative EU incorporation in irradiated and non-irradiated RPE1 Halo-HA-INTS11 KI cells, in which either WT or catalytic mutant (E203Q) was lentivirally expressed as indicated, 1 h and 22 h after UV exposure (8 J/m$^2$). Cells were treated with PROTAC3 (0.3 µM, 16 h). NT, not treated. Quantification is shown in Fig. 2E.

**G:** Representative images of transcription recovery by EU incorporation in Halo-HA-INTS11 KI cells and CSB KO cells after treatment with Illudin S (ILS, 75 ng/mL) at the specified time points. Where specified, cells were pre-treated with PROTAC3 (0.3 µM, 16 h). Illudin S was washed away with PBS and transcription was assessed at the indicated times after Illudin S treatment. Quantification is shown in Fig. 2F. NT, not treated and W.O., wash-out.

**H:** Immunoblot analysis for the indicated antibodies of ATF3 signal of lysates of RPE1 WT, STK19 KO and CSB KO cells before or 7, 24 or 32 h after UV treatment (8 J/m$^2$). BRG1 was used as a loading control.

## Supplemental Figure 3 Belonging to figure 3

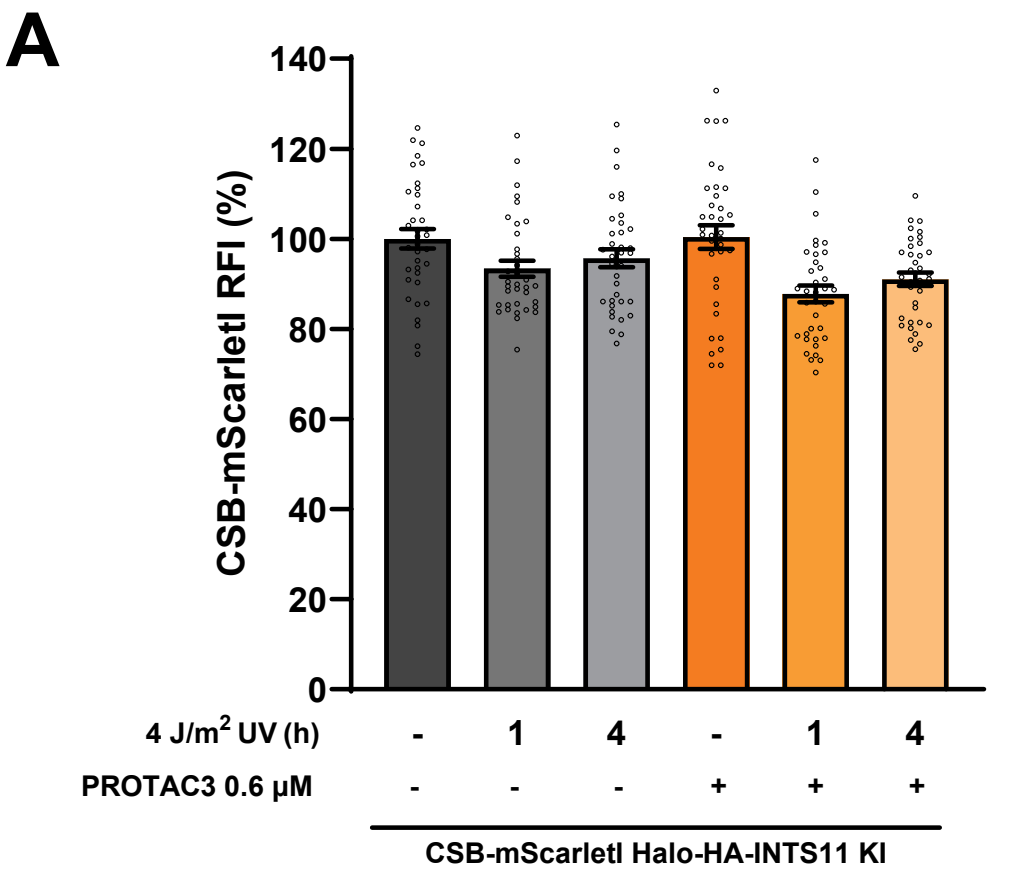


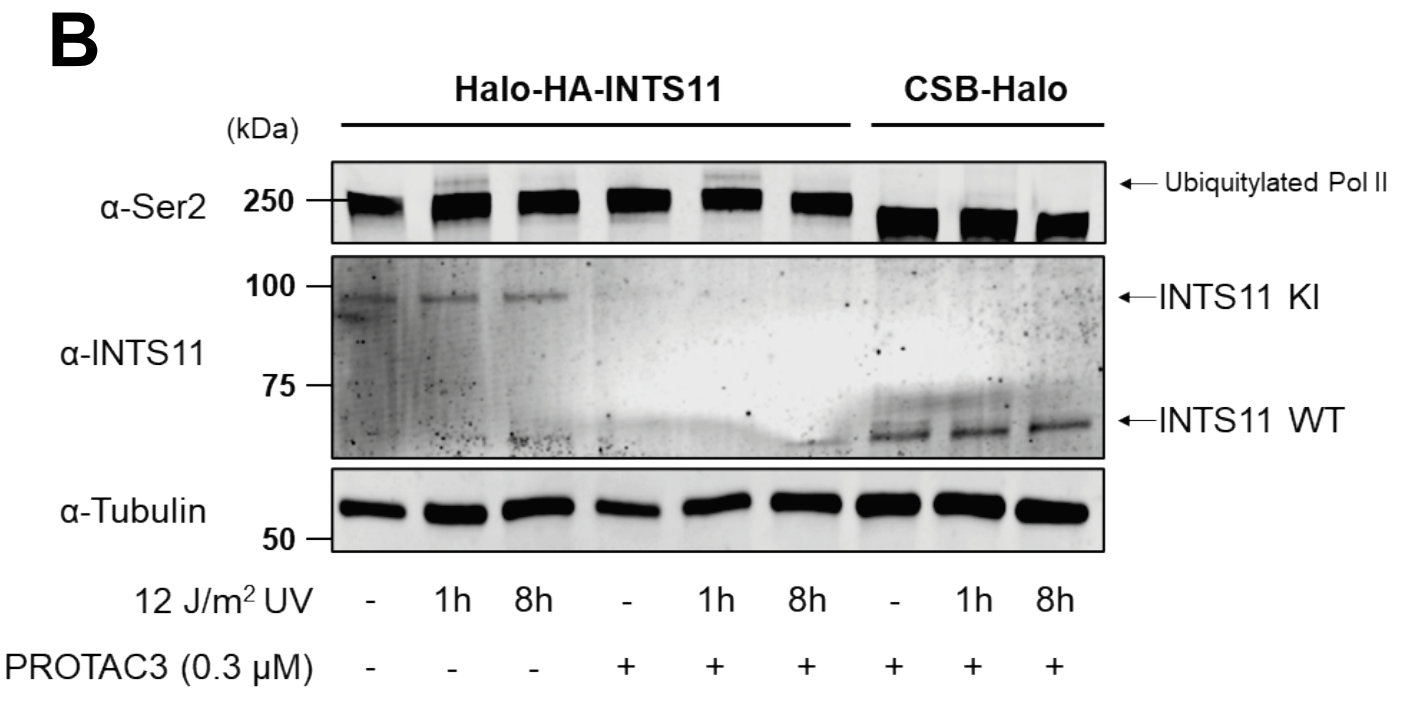


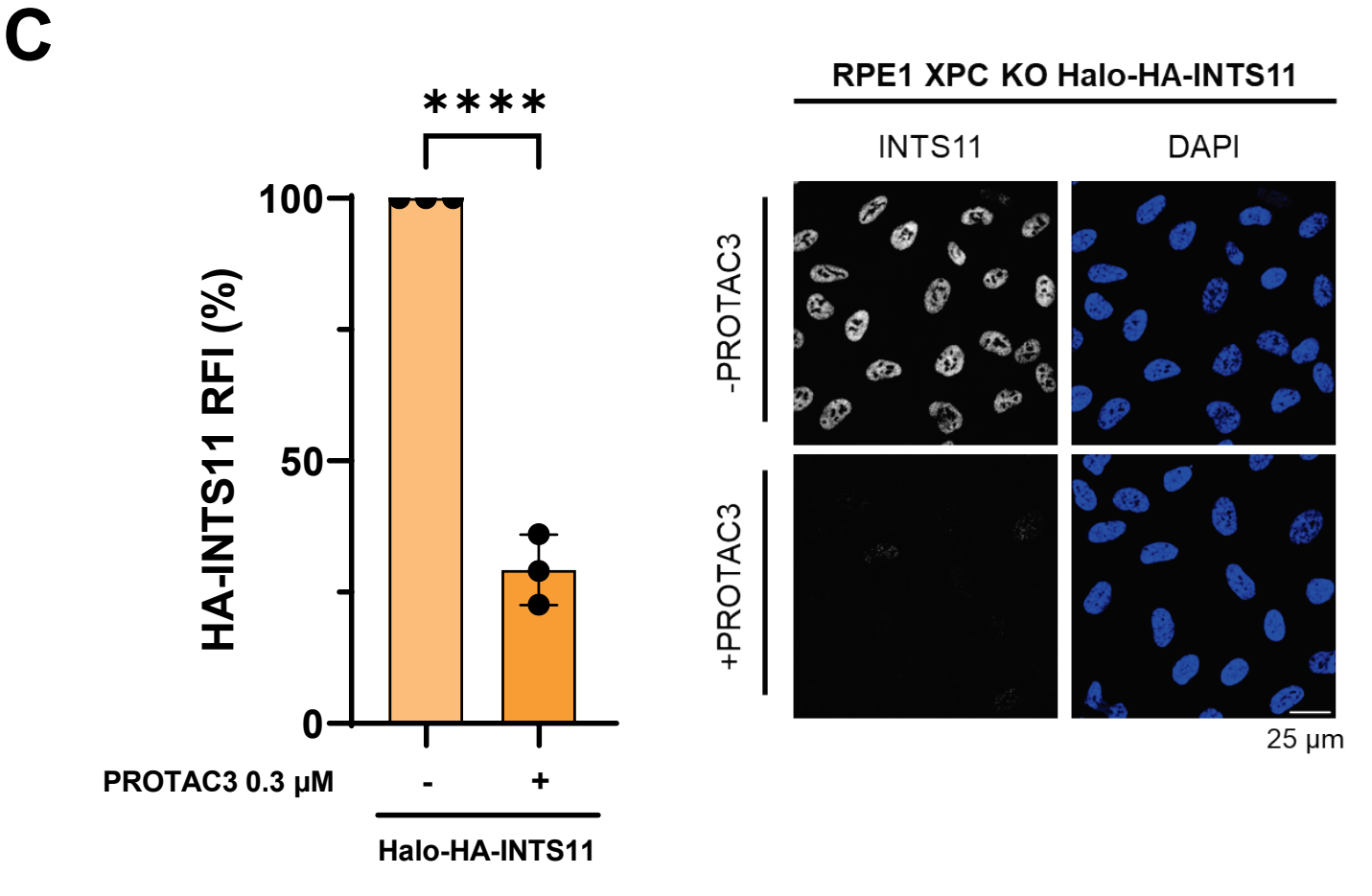


**A:** Quantification of CSB-mScarletI protein levels using relative fluorescence intensity (%) of cells analyzed by FRAP before photobleaching. Black lines indicate average integrated density ± SEM from three independent experiments (n = 36 cells per condition).

**B:** Immunoblot using the indicated antibodies of the chromatin fraction of HCT116 GFP-RPB1 and Halo-HA-INTS11 or CSB-Halo KI cells 1 h or 8 h after 12 J/m$^2$ UV or mock treatment. Where specified, cells were pre-treated with PROTAC3 (0.3 µM, 16 h) to deplete INTS11 or CSB. Tubulin was used as a loading control.

**C:** Quantification (Left) and representative images (Right) of INTS11 following treatment with PROTAC3 (0.6 µM, 24 h), as assessed by immunofluorescence of HA in RPE1 XPC-KO Halo-HA-INTS11 KI cells. IF signal was background corrected. Statistical significance was determined using an unpaired two-tailed Student's t-test (****$P < 0.0001$). These samples correspond to those used in TCR-UDS shown in Fig. 3E.

**Supplemental Figure 4 Belonging to figure 4**

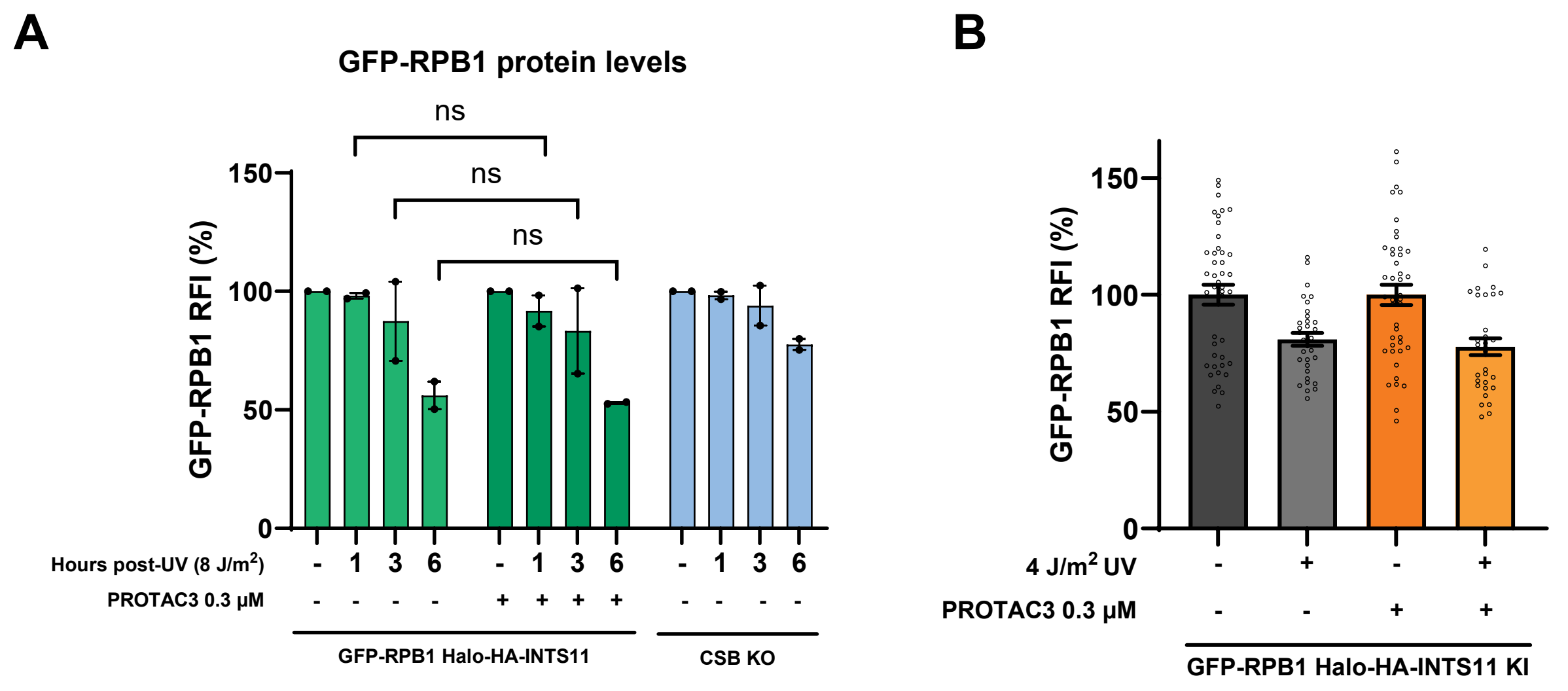


**A:** Pol II levels in RPE1 GFP-RPB1 Halo-HA-INTS11 KI and GFP-RPB1 KI CSB KO cells were quantified by flow cytometry. Where indicated, cells were pre-treated with PROTAC3 (0.3 µM, 16 h). Cells were mock-treated or irradiated with UV (12 J/m$^2$) and analyzed at the indicated time points. GFP-RPB1 levels in UV-irradiated cells were normalized to undamaged controls for each cell line (set at 100). Columns represent the mean relative fluorescence intensity (RFI) of two independent experiments of > 10.000 cells ± SEM. Statistical significance was assessed by an unpaired two-tailed Student's t-test (ns, non-significant).

**B:** Quantification of GFP-RPB1 protein levels using relative fluorescence intensity (%) of cells analyzed by FRAP before photobleaching. Black lines indicate average integrated density ± SEM from at least four independent experiments (n ≥ 31 cells per condition).

## Supplemental Figure 5 Belonging to figure 5

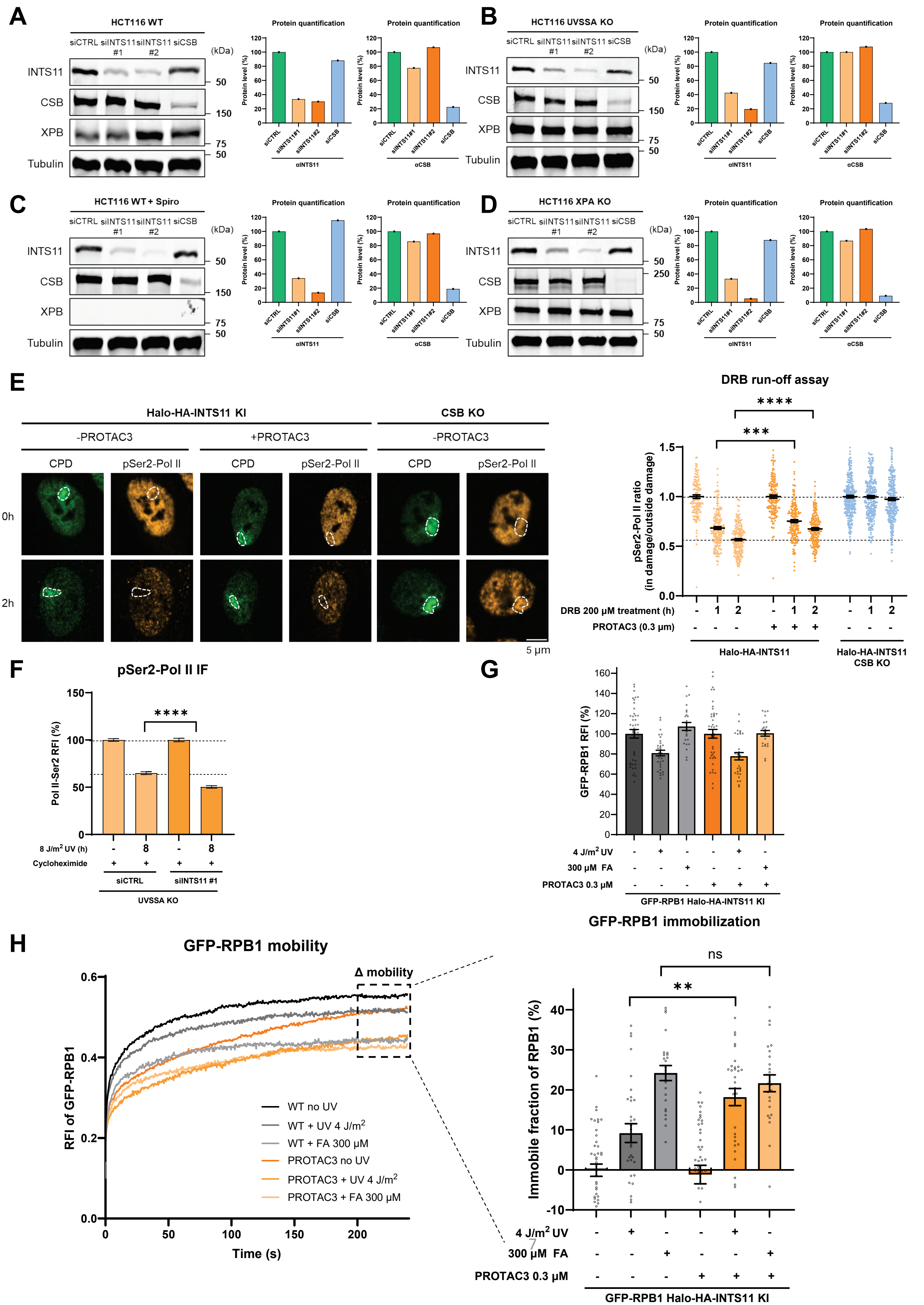

**Supplemental Figure 5 Belonging to figure 5**

**A:** Immunoblot (Left) and quantification (Right) of whole-cell lysates from HCT116 cells transfected with the indicated siRNAs to confirm efficient protein depletion. Protein levels were normalized to the loading control. Tubulin was used as a loading control and set at 100%. These samples correspond to those used in the experiment shown in Fig. 5B.

**B, C, D:** As in (A) in HCT116 (**B**) UVSSA KO, (**C**) spironolactone-treated WT (10 µM, 2 h) and (**D**) CSB-mScarletI KI XPA KO cells upon depletion of the indicated proteins using siRNAs. These samples correspond to those used in the experiment shown in Fig. 5C-E.

**E:** (Left) Representative single-cell images of pSer2-modified elongating Pol II at sites of local UV-induced DNA damage (white dash) as determined by IF 0 and 2 h after UV exposure (80 J/m$^2$). Where indicated cells were treated with PROTAC3 (0.3 µM, 16 h) to deplete INTS11. (Right) pSer2-modified elongating Pol II signal quantification at sites of local UV-induced DNA damage in RPE1 Halo-HA-INTS11 cells and RPE1 Halo-HA-INTS11 KI CSB KO. Where specified, cells were pre-treated with PROTAC3 (0.3 µM, 24 h) to deplete INTS11. Cells were locally irradiated with UV (80 J/m$^2$) and treated with DRB immediately following UV irradiation and IF was performed at the indicated times after UV. CPD staining was used to identify areas of local UV damage. The mean pSer2-modified Pol II intensity within the damaged region was divided by the mean intensity outside the damaged region in the same nucleus to normalize for the overall reduction in pSer2-modified Pol II signal caused by DRB-induced transcription inhibition resulting in a Pol II run-off. A ratio <1 indicates that pSer2-modified Pol II is released faster at sites of local damage than the general transcription run-off in undamaged regions. Black lines indicate average pSer2-Pol II ratio ± SEM from three independent experiments (n ≥ 140 cells per condition). Statistical significance was determined using an unpaired two-tailed Student's t-test (*** P < 0.001, **** P < 0.0001).

**F:** Quantification of pSer2-modified Pol II levels as determined by IF in HCT116 UVSSA KO cells transfected with the indicated siRNAs, either non-treated (NT) or 8 h after UV exposure (8 J/m$^2$). Cycloheximide (100 µM) was added 2 h prior to UV-irradiation and maintained for the duration of the experiment. The relative fluorescence intensity (RFI) was normalized to mock-treated levels and set at 100. Black lines indicate average RFI intensity and error bars ± SEM. n ≥ 247 cells per condition from three independent experiments. Statistical significance was determined using an unpaired two-tailed Student's t-test (****P < 0.0001).

**G:** Quantification of GFP-RPB1 protein levels using relative fluorescence intensity (%) of cells analyzed by FRAP before photobleaching. Black lines indicate average integrated density ± SEM from at least three independent experiments (n ≥ 24 cells per condition).

**H:** (Left) FRAP analysis of Pol II mobility in mock-, UV- (4 J/m$^2$, 1 h) and FA-treated (300 µM, 30 min) RPE1 GFP-RPB1 Halo-HA-INTS11 KI cells. PROTAC3 (0.3 µM, 24 h) pre-treatment was done when indicated. GFP-RPB1 relative fluorescence intensity (RFI) was background-corrected and normalized to pre-bleach fluorescence intensity and set to 1. n ≥ 24 cells per condition from at least three independent experiments. (Right) Calculated immobile fraction of GFP-RPB1 after DNA damage obtained from the data points in the dotted box indicated in left (timepoints 199.4 s to 239.4 s). Immobile fraction in WT and INTS11-depleted cells was calculated by comparing RFI after UV or FA to mock-treated sample and normalizing to their own non-irradiated RFI values. n ≥ 24 cells per condition from at least three independent experiments ± SEM. Statistical significance was determined using an unpaired two-tailed Student's t-test (**P < 0.01, ns, non-significant).
